# Inferring the physical properties of yeast chromatin through Bayesian analysis of whole nucleus simulations

**DOI:** 10.1186/s13059-017-1199-x

**Published:** 2017-05-03

**Authors:** Jean-Michel Arbona, Sébastien Herbert, Emmanuelle Fabre, Christophe Zimmer

**Affiliations:** 1 Unité Imagerie et Modélisation, Institut Pasteur, 25 rue du Docteur Roux, 75015 Paris, France; 2 UMR 3691, CNRS; C3BI, USR 3756, IP CNRS, Paris, France; 3 Université Paris Diderot, Sorbonne Paris Cité, Cellule Pasteur, 75015 Paris, France; 4 Chromosome Biology and Dynamics, Hôpital Saint Louis, Paris, France

**Keywords:** Chromatin, Chromosomes, Nuclear architecture, Polymer models, Yeast

## Abstract

**Background:**

The structure and mechanical properties of chromatin impact DNA functions and nuclear architecture but remain poorly understood. In budding yeast, a simple polymer model with minimal sequence-specific constraints and a small number of structural parameters can explain diverse experimental data on nuclear architecture. However, how assumed chromatin properties affect model predictions was not previously systematically investigated.

**Results:**

We used hundreds of dynamic chromosome simulations and Bayesian inference to determine chromatin properties consistent with an extensive dataset that includes hundreds of measurements from imaging in fixed and live cells and two Hi-C studies. We place new constraints on average chromatin fiber properties, narrowing down the chromatin compaction to ~53–65 bp/nm and persistence length to ~52–85 nm. These constraints argue against a 20–30 nm fiber as the exclusive chromatin structure in the genome. Our best model provides a much better match to experimental measurements of nuclear architecture and also recapitulates chromatin dynamics measured on multiple loci over long timescales.

**Conclusion:**

This work substantially improves our understanding of yeast chromatin mechanics and chromosome architecture and provides a new analytic framework to infer chromosome properties in other organisms.

**Electronic supplementary material:**

The online version of this article (doi:10.1186/s13059-017-1199-x) contains supplementary material, which is available to authorized users.

## Background

The mechanical properties of chromatin and the spatial arrangement of chromosomes play an important role in genome functions, but in general remain poorly known [1, 2]. The structure of the chromatin fiber has remained elusive and controversial. The classical 30 nm structure, in which nucleosomes are tightly stacked on top of each other, is now called into question [3–9]. Chromosome architecture has been intensely studied in recent years using genome-wide chromosome conformation capture (Hi-C), which provides precious quantitative information about chromatin folding and has revealed biologically important features such as promoter-enhancer interactions and chromosome partitioning into functional domains [10–15]. A key challenge is to build mechanistic models that are able not only to explain these observations, but also to predict the 3D organization of chromosomes and its alterations de novo. It is increasingly evident that polymer physics provides an adequate framework for this purpose [5, 16–26]. This is particularly clear for the extensively studied budding yeast nucleus [27–34]. We and others have shown that many aspects of yeast nuclear architecture can be reproduced in silico by modeling chromosomes as generic, semi-flexible polymers, with only a small number of sequence-specific constraints [35–37]. Such models can also make predictions on functional features, such as differences in DNA repair efficiency by homologous recombination [37, 38].

Nevertheless, two important questions remain unanswered. The first question relates to the mechanical and structural properties of the chromatin fiber, including its compaction and rigidity. The compaction *C* can be defined as the number of base pairs per unit length along the fiber (bp/nm). The bending rigidity can be measured by the persistence length *P*, the curvilinear distance along the fiber over which the direction (tangent vector) of the fiber becomes uncorrelated, such that more rigid fibers have higher *P*. Both parameters are key to the mechanical behavior of the chromatin during functional processes such as transcription and replication, but remain poorly characterized, and the very structure of chromatin remains uncertain. Previous estimates of *C* in yeast were in the range of 30–150 bp/nm and estimates of *P* were in the range of less than 30 nm to 200 nm or more [4, 12, 39–43].

Second, significant discrepancies still exist between model predictions and experimental observations [35]. If these discrepancies are due to errors in the model rather than the data, one must determine if they reflect incorrect values of the basic mechanical parameters of the model, including *P* and *C*, or rather reflect local effects possibly associated to a functional process, such as chromatin decondensation during transcriptional activation of a specific gene [27, 28, 44, 45]. Whereas the first type of discrepancy might be remedied simply by adjusting the global structural parameters, the second type of discrepancy calls for a more complex model, in which chromatin structure can change locally, e.g. depending on gene expression. Thus, correcting for global parameter mismatches will make it easier to identify discrepancies pointing to local modifications of chromatin architecture that may relate to biological functions.

In this paper, we present a computational framework that simultaneously addresses these two questions, using hundreds of polymer simulations with different chromatin parameters, combined with hundreds of experimental data points from multiple independent studies. Our analysis provides new constraints on the mechanical properties of the chromatin fiber, as well as a considerably more accurate predictive model of 3D nuclear architecture and chromatin dynamics in yeast.

## Methods

### Framework to infer chromatin parameters from whole-nucleus simulations

**Fig. 1:**
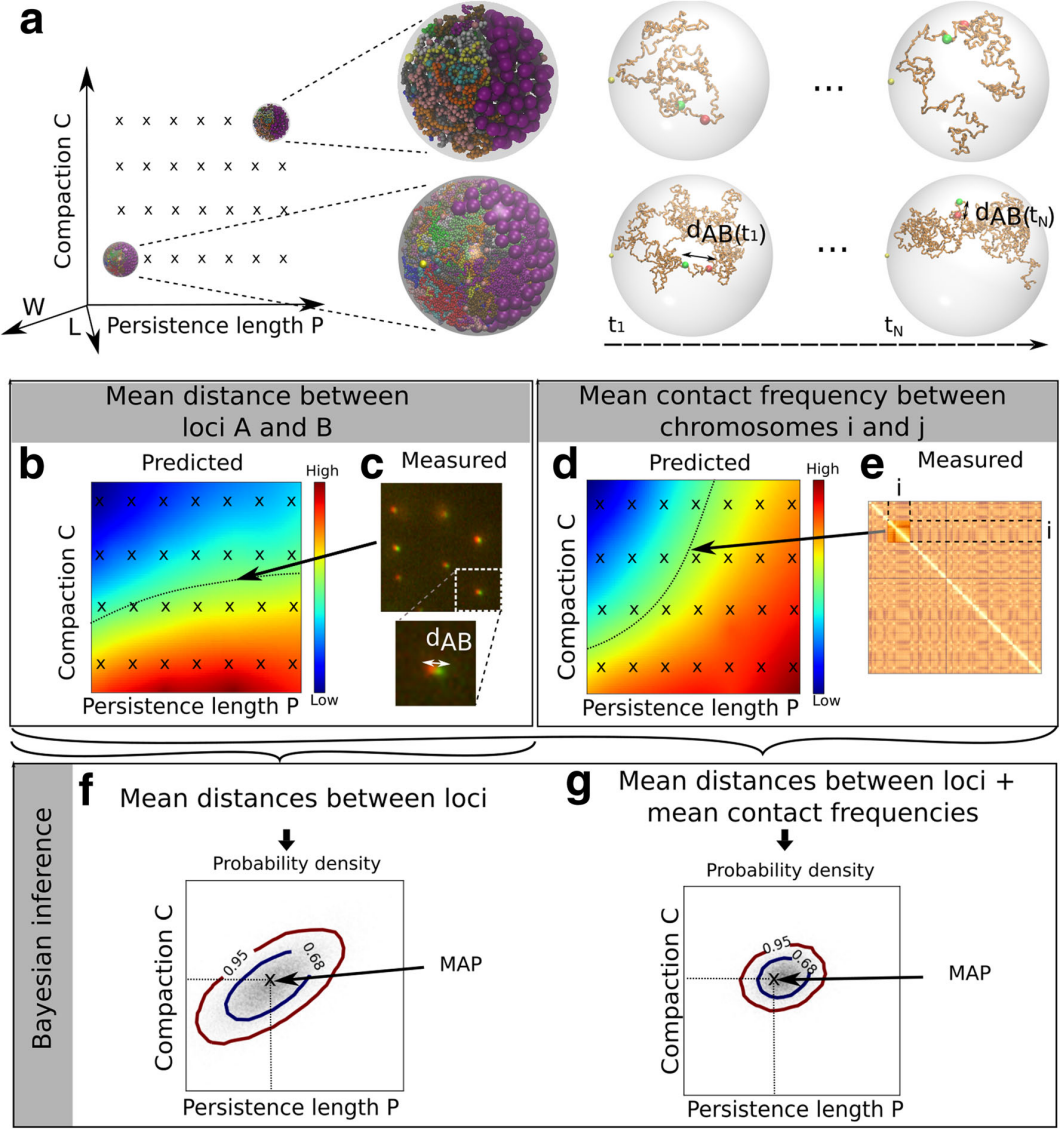
Main components of our computational framework for Bayesian inference of chromatin parameters from whole nucleus simulations. **a** Simulations: we consider a number n *=*144 of different parameter values Π_*i*_ = (*P*_*i*_, *C*_*i*_, *W*_*i*_, *L*_*i*_), where *P*_*i*_ is the chromatin persistence length, *C*_*i*_ the chromatin compaction, *W*_*i*_ the chromatin width, and *L*_*i*_ the length of microtubules (see Table 1, Additional file 2). The discretization of the parameter space is illustrated on the *left* (*crosses*), highlighting persistence length *P* and compaction *C*. Each Π_*i*_ defines a separate model *M*_*i*_ = *M*(Π_*i*_), for which we run two to six independent dynamic simulations of all 16 chromosomes in the nucleus with random initializations. Three-dimensional snapshots are shown for a model with high *P* and high *C* (*top*) and a model with low *P* and low *C* (*bottom*). Each simulation run calculates changes in chromosome configurations over millions of time steps, as illustrated for two time points *t*_1_ and *t*_*N*_ (only chromosome 4 is shown). By sampling these simulation runs, we predict various observables, ${Y}_k^{M_i}$, such as the average distance $\left\langle {\mathrm{d}}_{AB}^{M_i}\right\rangle$ between two loci A and B, or the average contact frequencies between chromosomes *i* and *j*. **b** For any value of the parameters Π (within the allowed range), an interpolation scheme calculates the predicted value of the observables *Y*_*k*_^*M*(Π)^, e.g. 〈d_*AB*_^Π^〉 shown here as a *heat map*, from the discrete models *M*_*i*_ (*crosses*). **c** Experimental data *Y*_*k*_^*E*^, such as the average distance between loci A and B measured by imaging, 〈d_*AB*_^*E*^〉, are compared to the predictions 〈d_*AB*_^Π^〉 for all Π. **d**, **e** Similarly, contact frequencies between chromosomes *i* and *j* are predicted for all Π (here *i*=*j*) (**d**) and compared to measurements from Hi-C experiments (**e**). **f**, **g** Parameter inference: given an experimental dataset, using the Bayes rule and Markov chain Monte Carlo sampling, we calculate the posterior probability density of any subset of parameters, such as (*P*, *C*). Isocontour lines enclose the region of high probability. This can be done for individual experimental data, e.g. 〈d_*AB*_^*E*^〉 (**f**), or for a combination of multiple datasets, e.g. mean distances between loci and chromosome contact frequencies (**g**). The maximum a posteriori estimate of the parameters (MAP) defines a model that provides the best match to the experimental data

**Fig. 2:**
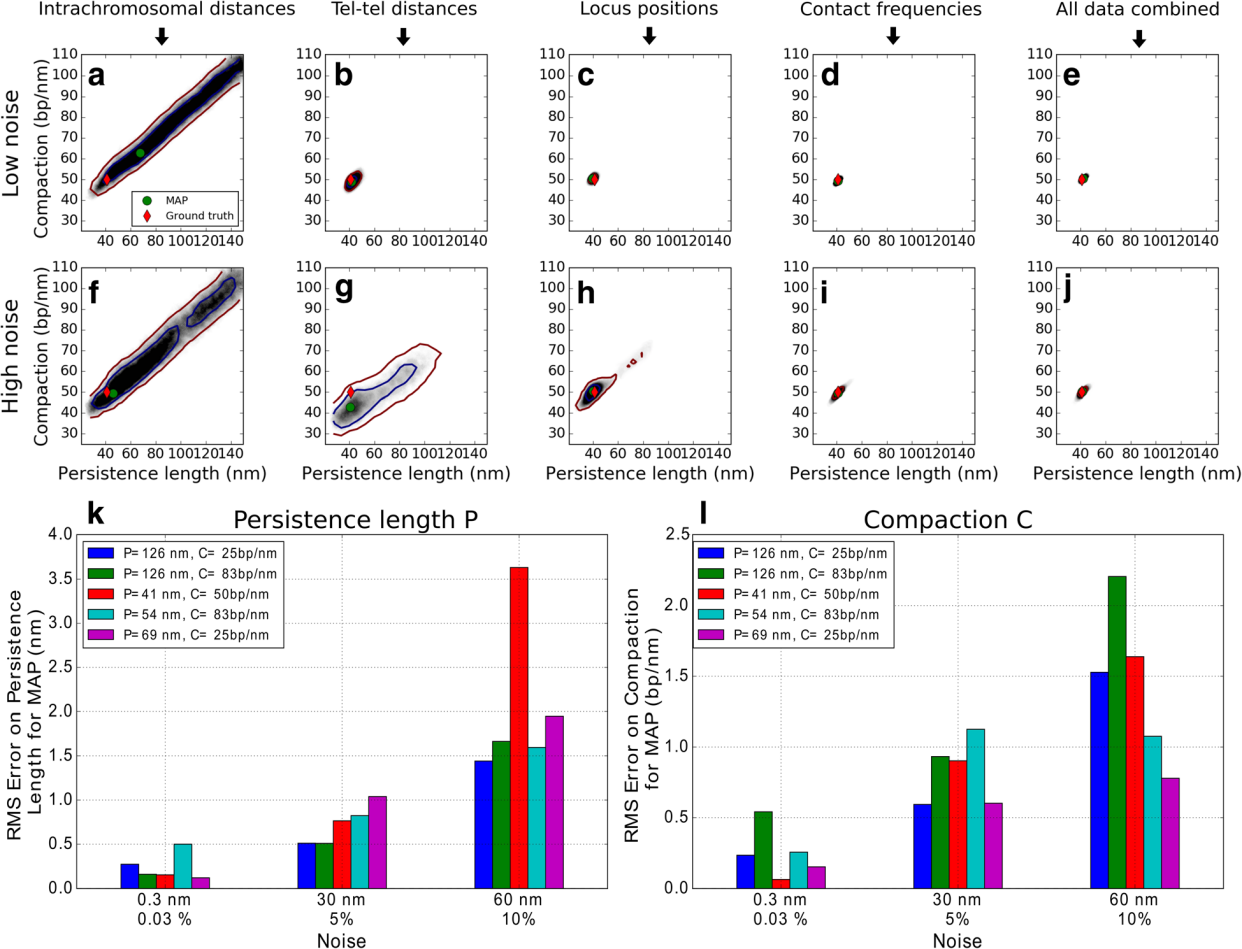
Validation of inference method on simulated data. This figure presents results of our parameter inference method when simulated data are used as input instead of experimental data. **a**–**j** Inferred posterior probability densities for chromatin compaction *C* and persistence length *P* for data generated by a model with parameters Π_0_ = (*P*_0_, *C*_0_, *W*_0_, *L*_0_) = (41 nm, 50 bp/nm, 45 nm, 300 nm). The *blue* and *red contour lines* enclose regions corresponding to 68% and 95% of the probability mass, respectively. The *red diamond* indicates the true parameter values: (*P*_0_, *C*_0_) = (41 nm, 50 bp/nm). The *green dot* indicates the maximum a posteriori (MAP) estimate, i.e. the parameter values $\left(\widehat{P},\widehat{C}\right)$ for which the estimated posterior probability density is maximum. Panels **a**–**e** were obtained from simulations with low levels of added noise, panels **f**–**j** from simulations with high noise (Additional file 1: Supplementary Methods). Panel pairs (**a**, **f**), (**b**, **g**), (**c**, **h**), (**d**, **i**), and (**e**, **j**) each correspond to a different subset of simulated observables. Panels **a**, **f**: probability densities obtained from distances between the pairs of loci corresponding to the experimental dataset O6 (see Table 2 and Additional file 3). Panels **b**, **g**: the same, for distances between pairs of telomeres (observable O1). Panels **c**, **h**: the same, for all locus positioning data, corresponding to observables O1–O7 combined. Panels **d**, **i**: the same, for contact frequencies between chromosomes (observables O8 or O9). Panels **e**, **j**: the same, for all distance and contact data combined (all observables, O1–O9). **k**, **l**: Errors of MAP estimates relative to the simulated ground truth for chromatin persistence length *P* (**k**) and compaction *C* (**l**). The root mean square (RMS) error is plotted for three different levels of noise and for five distinct simulated models (corresponding to five different values of the parameters), as indicated in the legend

**Fig. 3:**
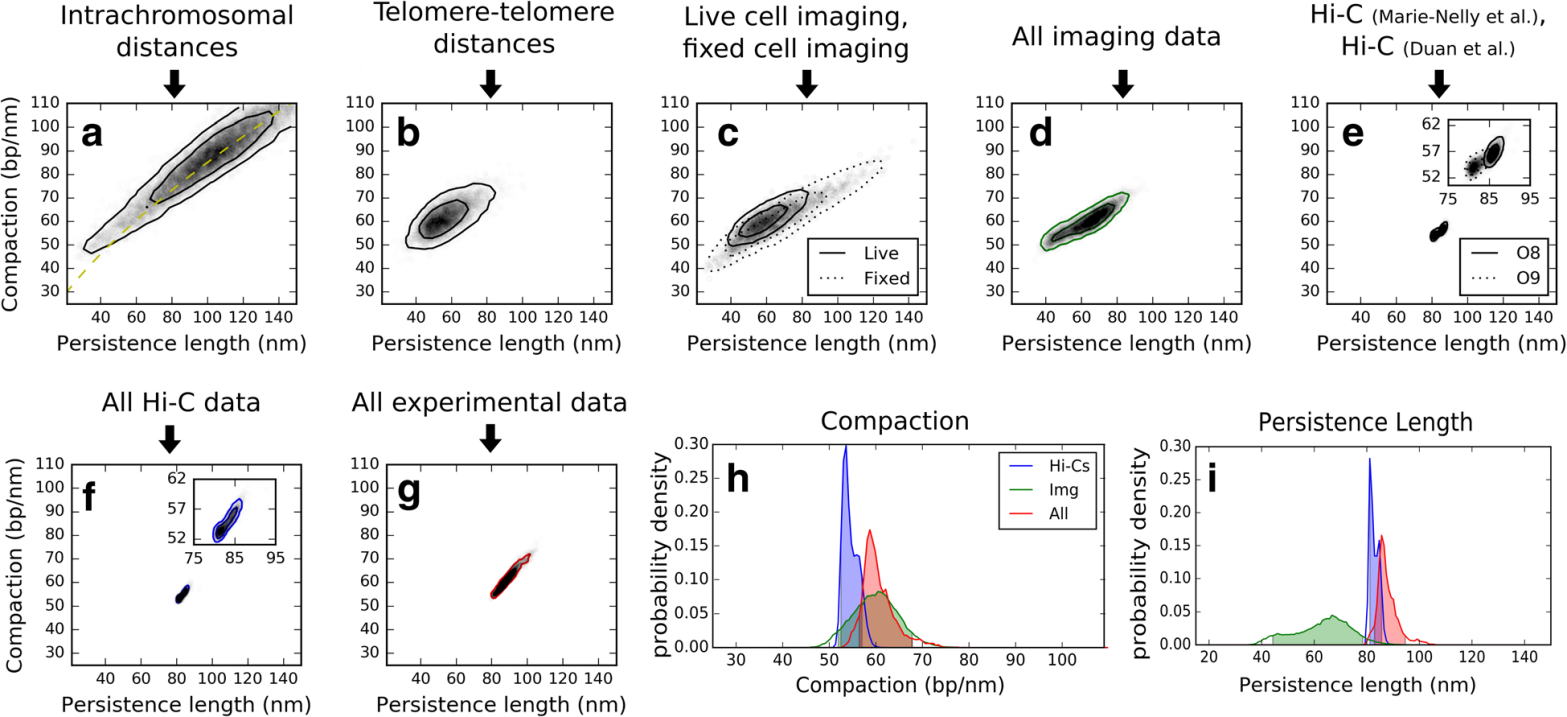
Yeast chromatin parameters inferred from imaging and Hi-C data. This figure shows the posterior probability densities of chromatin compaction *C* and persistence length *P* as inferred from a variety of experimental datasets. **a**–**g** joint posterior probability densities for (*P*, *C*) obtained for different subsets of experimental data (**a**–**f**) or the whole experimental dataset (**g**), as detailed below. The two *contour lines* shown enclose 68% and 95% of the probability mass. Individual panels correspond to the following experimental datasets: (**a**) modes of 3D distances between eight pairs of loci on chromosome 14 measured by imaging in fixed cells (observable O6, Table 2). The *dashed yellow curve* has *C* ∝ *P* ^0.7^. **b** Median 3D distances between 62 pairs of telomeres measured by imaging in live cells [50] (O1). **c** Combined set of data from imaging, in live cell or fixed cell experiments. Solid lines: combined data from live cell imaging (111 data points, O1–O4); dotted lines: combined data from imaging fixed cells (28 data points, observables O5–O7). **d** All imaging data from fixed and live cells pooled together (O1–O7; 139 data points). **e** Summary statistics from genome-wide contact frequencies measured by Hi-C data (see Table 2 and Additional file 1: Supplementary Methods). *Solid lines*: Hi-C data from [29] (56 data points; O8). *Dotted lines*: Hi-C data from [30] (56 data points; O9). **f** Combination of the two Hi-C datasets [29, 30] (116 data points; O8, O9). **g** Combination of all experimental data from imaging and Hi-C (266 data points; O1–O9). **h**, **i** Probability densities for *C* (**h**) and *P* (**i**), obtained either from all the imaging data as in (**d**) (*green*), from the two Hi-C datasets as in (**f**) (*blue*) or from the combination of imaging and Hi-C data as in (**g**) (*red*)

We begin with an overview of our analysis framework (Fig. 1). Briefly, we systematically varied the parameters Π of a whole nucleus chromosome simulation, which include the chromatin compaction *C* and the rigidity *P* (more parameters will be detailed below). We then aimed to determine the parameter values for which the model *M*(Π) agrees well with a range of experimental data and to quantify the plausibility of these parameters. The workflow comprises the following four components: (1) a set of hundreds of independent simulations of all chromosomes in the yeast nucleus, corresponding to 144 different parameter values Π_*i*_, (*i* = 1..144) (Fig. 1a); (2) nine sets of experimental data on nuclear architecture compiled from several distinct studies, each comprising many independent measurements Y_*k*_^*E*^ (*k =* 1…*N*, where *N* is the number of measurements and “*E*” stands for ‘empirical’), such as the mean distance between two chromatin loci (Fig. 1c) or the mean contact frequency between two chromosomes (Fig. 1e); (3) a module that, for any given parameter value Π, computes the model predictions Y_*k*_^*M*(Π)^ corresponding to these measurements, by interpolating the predictions from discrete parameter values Π_*i*_ (Fig. 1b, d); and (4) an algorithm that samples the parameter space and computes the posterior probability density of the parameters for a given dataset (Fig. 1f) or for multiple datasets taken together (Fig. 1g). We describe each of these components in more detail below.

### Whole-nucleus chromosome simulations and chromatin parameters

We simulated the spatial configurations and dynamics of chromosomes in the yeast nucleus using an approach similar to that described previously [35, 37] (see Additional file 1: Supplementary Methods for details). Briefly, our model simulated 16 randomly moving yeast chromosomes, each of which was represented as a chain of beads of diameter *W*, connected by non-linear spring potentials. The number of beads followed from the assumed compaction *C* and each chromosome’s genomic length (ranging from 230 Kb for chromosome 1 to 1531 Kb for chromosome 4). Triplets of consecutive beads were linked by a potential that penalizes bending and whose strength followed from the assumed persistence length *P*. The nucleus was modeled as a confining sphere of radius *R*_*N*_ = 1 μm. We included two additional constraints specific to the yeast nucleus. First, budding yeast centromeres are linked by a single microtubule to the spindle pole body (SPB), a macromolecular complex embedded in the nuclear envelope [27, 46]. We therefore introduced a harmonic (spring-like) potential between the centromeric bead of each chromosome and a point on the nuclear sphere representing the SPB, with an equilibrium length *L*. Second, telomeres are tethered to the nuclear envelope by two redundant pathways [27, 47]; we therefore applied a purely radial short-range outward force to the 32 telomeric beads to bring them in close vicinity to the nuclear envelope. Third, for the ~1–2 Mb chromosomal region encoding the ribosomal DNA (rDNA), we used beads of a diameter *W*_rDNA_ > *W*. This was done to account for the steric constraints exerted by the rDNA, which undergoes particularly intense transcription and gives rise to the nucleolus [27, 48]. A short-range repulsive potential prevented all beads and chromosome chains from traversing each other. Starting from an arbitrary initial configuration of all chromosomes within the nucleus (Additional file 1: Figure S1a, b), we used Langevin dynamics to simulate their movements, i.e. at each time step, each bead was subjected to a randomly oriented force, and its displacement was computed from the equations of motion resulting from this force and those derived from the abovementioned potentials. We let the simulation run for ~10^9^–10^10^ iterations to reach equilibrium and sampled the trajectories to predict observables *Y*_*k*_^*M*(Π)^ such as the mean distance between two loci or the average contact frequency between two chromosomes (Fig. 1b, d; Additional file 1: Figure S1c, d; Figure S2a, b).

Table 1 lists the main parameters used in our simulations, including those that had the same value for all simulations and those that we systematically varied, namely *P*, *C*, *W*, and *L*. We hereafter refer to the latter four parameters as “structural parameters” and note their combination Π = (*P*, *C*, *W*, *L*). In our previous study, we used Π_Wong_ = (*P*_Wong_, *C*_Wong_, *W*_Wong_, *L*_Wong_) = (30 nm, 83 bp/nm, 20 nm, 380 nm) based on the assumption of a compact fiber structure and early biophysical studies of chromatin reconstituted in vitro [35, 43, 49]. However, as already mentioned, the structural properties of the chromatin fiber remain largely undetermined. We therefore varied these parameters over the following ranges, which encompass most previous estimates [5, 39–43]: *P*: 27–252 nm, *C*: 25–110 bp/nm, *W*: 30–60 nm, *L*: 200–400 nm. We discretized the parameter space into 144 distinct values of Π_*i*_ = (*P*_*i*_, *C*_*i*_, *W*_*i*_, *L*_*i*_) (Additional file 2). For each Π_*i*_, we ran two to six independent (replica) simulations with random initialization, to assess the effect of purely statistical differences and check for equilibration. In total, we run 473 independent simulations, which we sampled every 10^4^ iterations, resulting in hundreds of millions of different chromosome configurations. We hereafter refer to these simulations as “core simulations.”

**Table 1:** Simulation

Parameter	Notation	Unit	Range	Wong 2012	MAP	Best model
Chromatin persistence length	*P*	nm	27–252	30	88	69
Chromatin compaction	*C*	bp/nm	25–110	83	61	50
Chromatin fiber width	*W*	nm	30–60	20	37	30
Microtubule length	*L*	nm	200–400	380	390	400
rDNA diameter	*W*_rDNA_	nm	194^a^	200	NA	194
Radius of nucleus	*R*_*N*_	nm	1000	1000	NA	1000

**Fig. 4:**
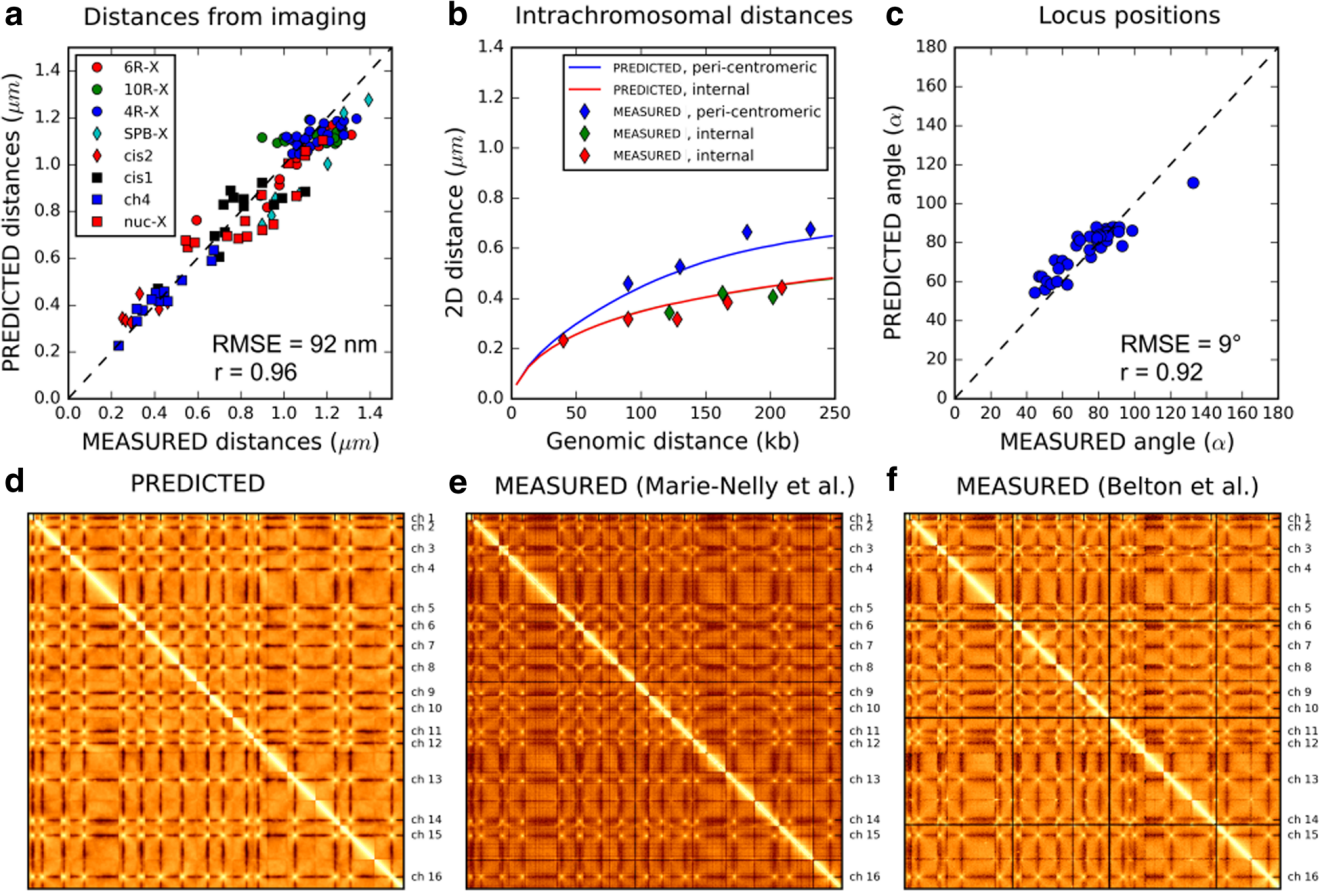
Comparing model predictions to static experimental data. This figure compares predictions from our simulation with the parameters *P* = 69 nm, C = 50 bp/nm, W = 30 nm, L = 400 nm (“best model”, Table 1) to different experimental data. **a** predicted vs measured distance statistics between pairs of loci (Table 2, observables O1, O3–O7). Each of the 117 dots corresponds to a different pair of loci. *Blue circles*: distances between telomere 4R and other telomeres [50]; *green circles*: distances between telomere 10R and other telomeres [50]; *red circles*: distances between telomere 6R and other telomeres [50]. *Blue squares*: distances between pairs of loci on chromosome 4. *Cyan diamonds*: distances between SPB and telomeres [39]. *Red diamonds*: intrachromosomal distances for pairs of loci on chromosomes 6 and 14 [39]. *Black squares*: intrachromosomal distances for pairs of loci on chromosomes 4, 5, and 7 [51]. *Red squares*: distances between loci on chromosome 12 and the nucleolar center [64]. The Pearson correlation coefficient between predictions and measurements is *r* = 0.96 and the RMS error is 92 nm. **b** Predicted and measured median 2D distances between 12 pairs of loci on chromosome 4 as function of their genomic separation (in Kb). *Diamonds* show experimental measurements, *solid curves* are model predictions. *Blue dots* are for pairs of loci involving a pericentromeric locus (5 Kb from the centromere). *Green* and *red dots* are for pairs of loci involving a locus in the internal region of the chromosome arm (at 854 Kb and 1185 Kb from the centromere, respectively). The *solid blue curve* shows the predicted distance between the peri-centromeric locus and other loci on the same chromosome arm. The *red curve* shows the predicted distance between loci in the internal region of the chromosome arm. **c** Predicted vs. measured median angle (in degrees) between chromatin loci and the line joining the nuclear and nucleolar centers [31, 50]. Each *dot* corresponds to a single chromatin locus. The Pearson correlation between predictions and measurements is *r* = 0.92 and the RMS error is 9 degrees. **d**–**f** Genome-wide contact frequency matrices, binned at 30 Kb, as predicted by the model (**d**) or obtained from Hi-C experiments in [30] (**e**) and [62] (**f**). *Bright pixels* indicate high frequencies, *dark pixels* indicate low frequencies. A logarithmic scaling was applied to reveal lower frequency contact patterns

**Fig. 5:**
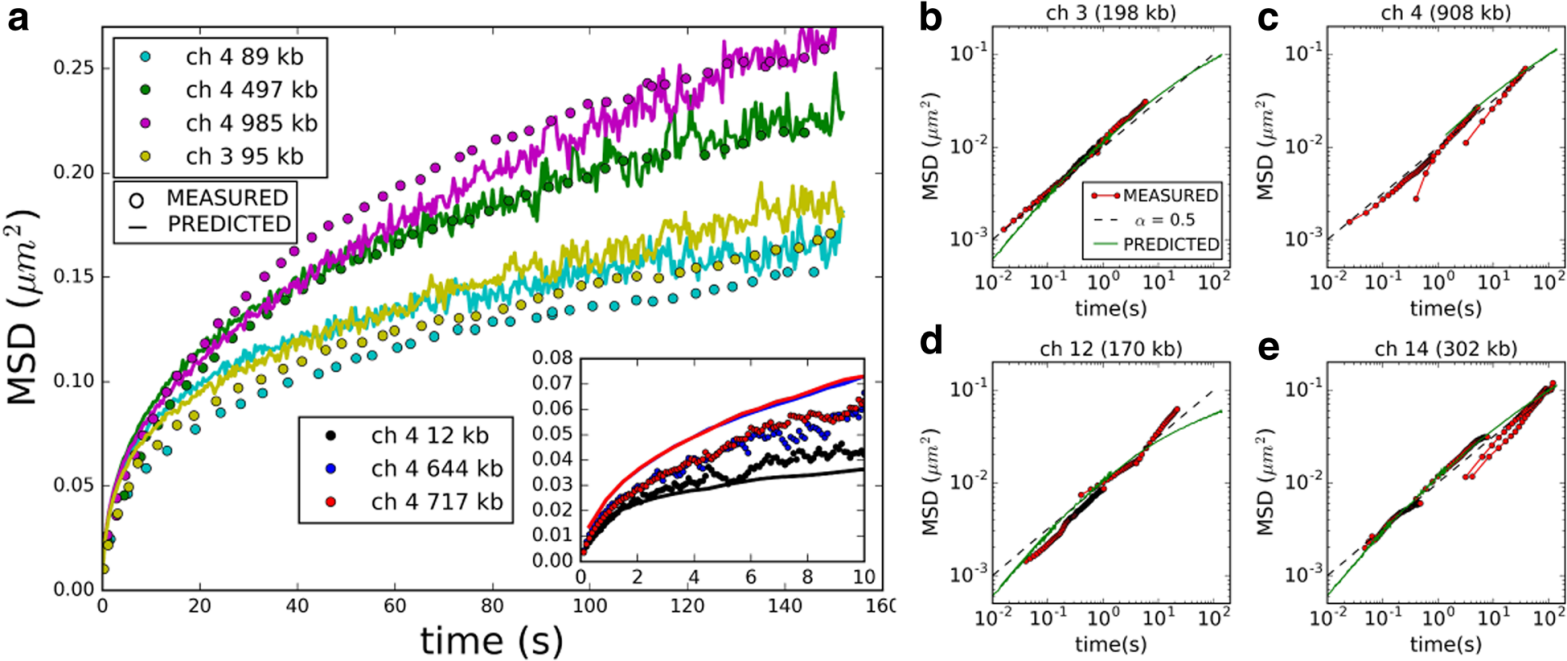
Comparing model predictions to chromatin dynamics data. This figure shows predicted (*solid lines*) and measured (*dots*) mean-square displacements (MSD) of single chromatin loci as function of time interval. **a** MSD of six loci on chromosome 4 and the MAT locus on chromosome 3, over time intervals of 1–150 s (main plot, data from [65]) or 1–10 s (inset, data from [66]). For each of the two datasets, a single time step parameter was fitted once to align simulation time with experimental time. The genomic distance of each locus to the centromere is indicated in the legend. **b**–**e** MSD of four loci on four different chromosomes, over time intervals in the range of 16 ms to 100 s, on a double logarithmic scale. The chromosome number and the genomic distance of the locus to the centromere are indicated on top of each panel. Data (*dots*) are measurements from time-lapse microscopy in [64]. *Green curves* are model predictions. *Dashed black lines* show a subdiffusive power law MSD ∝ *t* ^0.5^ as expected from the Rouse model [22, 37, 41]

This table lists the main parameters used in our model (first column), together with their notation (second column), and units (third column). The fourth column indicates the range over which the parameters are varied (for parameters that are not varied but held fixed in all simulations, a single value is given). The fifth column indicates values used in our previous model by Wong et al. [35]. The sixth column indicates the maximum a posteriori (MAP) estimate of each inferred parameter as obtained by our method using the whole set of experimental data. The seventh column lists the parameters of the “best model” used for Figs. 4 and 5. NA: not applicable. Additional file 2 lists all 144 models and their parameter values. More details on model parameters are provided in Additional file 1: Supplementary Methods

^a^This diameter corresponds to a net volume of the 150 rDNA beads that equals *V* = 14% of the nuclear volume, i.e. an effective volume (considering free space between rDNA beads) of ~2 V ~30% of the nucleus. For the parameter mismatch analysis, *W*_rDNA_ was varied over a range of 138–251 nm, corresponding to a net volume fraction *V* = 5–30%, and an effective fraction of ~2 *V* = 10–60%

### Compilation of experimental data on yeast chromatin architecture 

We gathered a large amount of quantitative measurements on yeast nuclear architecture based on light microscopy in live or fixed cells and on Hi-C. The microscopy data contained mostly distances between pairs of loci on the same chromosome (*cis*) or on different chromosomes (*trans*), which were typically measured in hundreds or thousands of individual cells [35, 39, 50, 51]. We also added newly measured distances between 12 pairs of loci along the right arm of chromosome 4, spanning pericentromeric, internal, and subtelomeric regions (Additional file 1: Supplementary Methods). The Hi-C data provided genome-wide maps of contact frequencies between DNA segments averaged over millions of cells [29, 30]. Most of the imaging and Hi-C data were acquired and quantified by independent groups [29, 30, 35, 39, 50, 51]. While these data contain many thousands of individual measurements, we chose to condense them into a more manageable set of summary statistics. For example, instead of the entire distribution of distances between two loci, we only considered the mean (or median) distance over all cells. Similarly, we reduced the Hi-C data to the following quantities: (1) average contact frequencies of each chromosome with itself; (2) average contact frequencies of each chromosome with all other chromosomes; (3) intrachromosomal contact frequencies for different genomic distances, averaged over the entire genome; (4) intrachromosomal contact frequencies relative to the centromere, averaged over all chromosomes. Table 2 summarizes the experimental datasets and summary statistics. Additional file 3 provides a detailed list of all image-based measurements we have used. In the following, we separately pooled image-based measurements from fixed cells (28 data points *Y*_*k*_^*E*^) and live cells (126 data points) because of the potentially important effect of fixation on nuclear architecture. We also considered the two Hi-C studies [29, 30] separately because of potentially important differences in protocols and data analysis (56 data points each). In total, our compilation thus consisted of 266 independent data points *Y*_*k*_^*E*^, hereafter also called “observables.” See Additional file 1: Supplementary Methods for more details.

**Table 2:** Experimental data used for parameter inference

Dataset	Observable	Experimental technique	Data points (n)	Reference
O1	Median 3D distance between pairs of subtelomeric loci	Imaging, live cells	62	[50]
O2	Median angle between locus, nuclear and nucleolar centroids	Imaging, live cells	37	[31, 50]
O3	Median distance between chromosome 12 locus and nucleolus	Imaging, live cells	15	[64]
O4	Median 2D distance between pairs of loci on chromosome 4	Imaging, live cells	12	this study
O5	Mean 3D distances between intrachromosomal pairs of loci on several chromosomes	Imaging, fixed cells (FISH)	13	[51]
O6	Mode of 3D distances between intrachromosomal pairs of loci on chromosome 14	Imaging, fixed cells (FISH)	8	[80]
O7	Mode of 3D distances between SPB and telomeres	Imaging, fixed cell (Immunofluorescence)	7	[80]
O8	Mean contact frequencies within chromosomes (16) and of each chromosome with the other chromosomes (16) + Intrachromosomal contact frequencies for genomic distances 25–85 Kb at 5 Kb intervals, averaged over the genome (12) + relative to the centromere (12)	Hi-C	56	[29]
O9	Same	Hi-C	56	[30]
	Total		266	

This table summarizes the nine experimental datasets used for parameter inference in this study. Additional data used for model validation only are: Hi-C data from [62], Micro-C XL data from [63], and chromatin dynamics data from [64–66]. For more details, see Additional file 3 and Additional file 1: Supplementary Methods

### Determining the probability density of chromatin parameters by Bayesian inference

We aimed to explore the space of chromatin parameters Π and to assign a probabilistic score to all possible values of Π based on how accurately the corresponding model *M*(Π) predicted a given experimental dataset *D*. This approach is more informative than an optimization method, which would only determine a single parameter value Π*, irrespective of how well the model explains the data for Π ≠ Π*. Our method employed two main ingredients: (1) a Bayesian formulation that computes the (posterior) probability of an assumed parameter value Π given the data *D*; and (2) a Monte Carlo Markov Chain algorithm that samples the space of parameters Π.

The Bayes rule provides the posterior probability of Π given *D* by: *p*(Π|*D*) ∝ *p*(*D*|Π)*p*(Π), where *p* indicates probability density and *p*(*A*|*B*) the probability density of *A* conditioned on *B*. Let us consider an experimental dataset *D* = (*Y*_1_^*E*^, …, *Y*_*N*_^*E*^) consisting of *N* independent measurements *Y*_*k*_^*E*^. For example, *Y*_*k*_^*E*^ might represent the mean distance between two loci A and B (Fig. 1c). For each data point *Y*_*k*_^*E*^, and for a given parameter value Π, the corresponding model *M*(Π) provides a single predicted counterpart *Y*_*k*_^*M*(Π)^ (Fig. 1b, d). Both the experimental measurements *Y*_*k*_^*E*^ and the model predictions *Y*_*k*_^*M*(Π)^ are, however, affected by noise: distances measured from images are necessarily corrupted by random localization errors [50, 52, 53], while Hi-C data suffer from counting noise due to limited sequencing depth, random ligations, and other factors [54, 55]. Our model predictions are also subject to random errors because they are computed from finite samples of stochastic simulations (Fig. 1a). For simplicity, we assume that the difference between each measurement *Y*_*k*_^*E*^ and the corresponding predictions *Y*_*k*_^*M*^ in the ideal case (i.e. assuming that the model faithfully describes reality) obeys a Gaussian probability density with variance *σ*_*k*_^2^. The variances *σ*_*k*_^2^ are usually not known. Depending on the type of data *Y*_*k*_^*E*^, they can be expressed as functions of one or more additional parameters Ξ (nuisance parameters), which must be estimated along with the structural parameters Π [56, 57]: *p*(Π, Ξ|*D*) ∝ *p*(*D*|Π, Ξ)*p*(Π)*p*(Ξ) (where we assumed statistical independence of Ξ and Π). For the structural parameters Π, we assumed flat priors, i.e. constant *p*(Π) over the parameter range mentioned above; the priors *p*(Ξ) for the nuisance parameters were different for each observable. See Additional file 1: Supplementary Methods for details. Thus, for any assumed value of the structural and nuisance parameters Π and Ξ, and for a given dataset *D*, the above formulation yielded a posterior probability *p*(Π, Ξ|*D*) (to within a normalization constant).

In order to estimate *p*(Π, Ξ|*D*) over the entire parameter space, we used a Monte Carlo Markov Chain ensemble sampler that efficiently concentrated the exploration of the high-dimensional parameter space (Π, Ξ) to the region of high posterior probability [58]. The posterior probability over the structural parameters, *p*(Π|*D*) was then readily obtained by integrating *p*(Π, Ξ|*D*) over the nuisance parameters Ξ. Similarly, the posterior probability for a single parameter, e.g. chromatin compaction, *p*(*C*|*D*), or the joint probability for a subset of parameters, e.g. *p*(*P*, *C*|*D*), were obtained by integrating over the remaining parameters (Fig. 1f, g). From the joint posterior probability density, we also determined a single maximum a posteriori (MAP) estimate of the parameters as: $\left(\widehat{P},\widehat{C}\right)= \arg \max p\left( P, C\Big| D\right)$ (Fig. 1f, g).

## Results

### Inference method recovers true chromatin parameters from noisy simulated data

To assess our method’s ability to recover the correct parameter values Π, and to better determine how different observables depend on these parameters and can be used to infer them, we first tested our method on synthetic data. To create these synthetic data, we picked a parameter value ${\Pi}_0={\Pi}_{i_0}$ among those of our core simulations (*i*_0_ ∈ [1, 144]) (Table 1 and Additional file 2); from the corresponding simulation ${M}_0={M}_{\Pi_0}$, we then computed predictions ${Y}_k^{M_0}\left( k=1..266\right)$ corresponding to all 266 observables mentioned above (Table 2). We then added random noise to these predictions to simulate experimental errors: ${Y}_k^S={Y}_k^{M_0}+{\varepsilon}_k\left( k=1..266\right)$, where *ε*_*k*_ is a normally distributed random number with mean 0 and variance (*σ*_*k*_^*S*^)^2^ (the superscript “S” denotes “simulation”). We tested three different levels of noise, with the highest level chosen such as to be consistent with or exceed the noise in the experimental data (Additional file 1: Supplementary Methods). We then used the noisy simulated data *D* ^*S*^ = *Y*_*k*_^*S*^(*k* = 1..266) instead of the real data as input to our inference algorithm (Fig. 1c, e) and compared the inferred posterior probabilities (Fig. 1f, g) to the true parameter value Π_0_. We performed this comparison for five different values of Π_0_, chosen near the boundaries of the explored parameter space (see Additional file 1: Figure S3).

Figure 2a–j shows *p*(*P*, *C*|*D* ^*S*^), the joint posterior probability for chromatin compaction *C* and rigidity *P*, computed for different subsets of observables *D* ^*S*^ or all observables together, for Π_0_ = (*P*_0_, *C*_0_, *W*_0_, *L*_0_) = (41 nm, 50 bp/nm, 45 nm, 300 nm), and assuming either low (Fig. 2a–e) or high levels of noise (Fig. 2f–j). These plots highlight how different observables constrain chromatin compaction and rigidity. For example, it is apparent from the elongated probability density in Fig. 2a that intrachromosomal distances can be used to determine *C* if *P* is known, or vice versa, but do not suffice to determine both parameters simultaneously. This is consistent with the well-known behavior of ideal or real polymer chains, where the mean square distance 〈*R* ^2^〉 between two loci separated by genomic distance *s* depends on *P* and *C* only via their combination *P* ^1 − *υ*^/*C* ^*υ*^ through the relation 〈*R* ^2^〉 ∝ (*P* ^1 − *υ*^/*C* ^*υ*^)^2^ *s* ^2*υ*^, (where *υ* = 0.5 and *υ* = 0.6 for an ideal and real chain, respectively [59]). However, our analysis can also reveal less obvious constraints. For example, Fig. 2b shows that unlike intrachromosomal distances, distances between telomeres do in fact allow to simultaneously determine *P* and *C*. The same holds true for contact frequencies between chromosomes (Fig. 2d). The probability densities obtained by combining all distances, or all distances and contact frequencies are shown in Fig. 2c and e, respectively. For low levels of noise, the computed posterior probability density based on the combined data is sharply peaked at the true parameter value Π_0_ (Fig. 2e). Picking the MAP parameters, we obtain an accurate match to the true values *C*_0_ and *P*_0_, with root mean squared (RMS) errors < 1 nm for *P* and < 1 bp/nm for *C*, based on six independent simulations, and for each of the five parameter values Π_0_ (Fig. 2e, k, l). At the highest level of noise we considered, the posterior probability densities *p*(*P*, *C*|*D* ^*S*^) broadened for all sets of observables (Fig. 2f–j), as expected, implying that the parameters were less well constrained by the data, but still contained the true value (*P*_0_, *C*_0_) within the 95% percentile region. This was true for all five parameter values Π_0_ tested. We further quantified the MAP estimation error as function of noise (Fig. 2k, l). While the RMS error increased with noise, as expected, we found that it always remained < 4 nm for *P* and < 2.5 bp/nm for *C* (Fig. 2k, l). We obtained similar errors, when instead of using a core simulation to generate the noisy input data, we used an independent replica that had not been employed for the interpolation in Fig. 1 (for (*P*_0_, *C*_0_) = (41 nm, 50 bp/nm)) (Additional file 1: Figure S4a, b, “replica”).

Thus, realistic levels of noise in the experimental data should entail only moderate errors in determining chromatin compaction and rigidity by our method.

### Chromatin parameter inference is robust to moderate model mismatch

To further assess the robustness of our parameter inference method, we tested the effect of a model mismatch, i.e. when the data are not strictly consistent with any of the assumed models (even discounting noise). To do this, we generated synthetic data from a new simulation with parameter values Π outside of the range explored by our core simulations. Specifically, we increased or decreased the parameter *W*_rDNA_ that specified the diameter of the chromosome region representing the rDNA, in such a way that the net nucleolar volume *V* changed from *V* = 14% of the nuclear volume (its value in all core simulations) to either *V* = 5%, 10%, 16%, 20%, or 30% (Table 1). In absence of noise, the MAP estimation of *P* and *C*, as computed from the combined data, fell within 7 nm and 11 bp/nm of the ground truth (RMS error), respectively, except for the simulation with *V* = 30% (Additional file 1: Figure S4a, b). In presence of added noise, the RMS errors of MAP estimations increased, but remained within 16 nm and 10 bp/nm even for high noise, again except for *V* = 30% (Additional file 1: Figure S4a, b). For *V =* 30%, errors became unacceptably high (≈40 nm and ≈ 30 bp/nm for *P* and *C*, respectively) (Additional file 1: Figure S4a, b). However, this case corresponds to a nucleolus effectively occupying almost 2 *V* = 60% of the nuclear volume, which is unreasonably large and leads to a considerably degraded agreement of model predictions with the experimental data (Additional file 1: Figure S4c).

These results suggest that our method to infer mechanical chromatin parameters is robust both to realistic levels of noise and to reasonable parameter mismatch.

### New constraints on mechanical chromatin properties

We then applied our inference method to the experimental data described above (Table 2 and Additional file 3). The posterior probability densities for persistence length *P* and compaction *C* estimated by our method are shown in Fig. 3, for different subsets of the data (Fig. 3a–f), and all datasets combined (Fig. 3g). Intrachromosomal distances alone constrained the compaction *C* and persistence length *P* to the vicinity of a curve given by: *C* ∝ *P* ^0.7^ (Fig. 3a), but these data could not separately determine *C* and *P* on their own, as expected from the above simulation results (Fig. 2a). By contrast, also as expected (Fig. 2b), distances between telomeres allowed to constrain both parameters to a much smaller range, namely *C* = 61 ± 6 bp/nm and *P* = 56 ± 10 nm (mean ± standard deviation) (Fig. 3b). When all imaging data were combined, a similar posterior probability density was obtained, with *C* = 60 ± 5 bp/nm and *P* = 63 ± 11 nm (Fig. 3d). Interestingly, imaging data obtained in live and fixed cells resulted in similar probability densities, despite the potential artefacts caused by fixation [50, 60] (Fig. 3c).

We next analyzed two independent Hi-C datasets [29, 30] and found that they again led to very similar probability densities (Fig. 3e). This is remarkable, given that these data were obtained using different experimental protocols and that both led to rather narrow densities. The probability density obtained when combining both Hi-C datasets is shown in Fig. 3f. Although the joint density obtained from Hi-C does not strictly overlap with that determined by imaging, it is relatively close (Fig. 3d, f). For the compaction *C*, the posterior density derived from the Hi-C data is 55 ± 2 bp/nm and overlaps substantially with that derived from the imaging data (Fig. 3h). For the persistence length *P*, the Hi-C data yielded an estimate of *P =* 83 ± 2 nm, an approximately 30% increase relative to the range *P =* 63 ± 11 nm determined by imaging (Fig. 3i). This discrepancy can potentially arise from multiple sources, including estimation errors due to mismatch of other parameters (see above), measurement errors exceeding our estimates, differences in data processing, or biological differences related to the different experimental protocols used in imaging or Hi-C. For example, a fixation-induced shrinkage of the nucleus without alteration of chromatin properties should result in an underestimation of compaction and an overestimation of persistence length. This might explain why the Hi-C data alone predicted lower *C* and higher *P* than the imaging data alone (Fig. 3h, i). If we ignored these discrepancies and combined all 266 experimental data points, we obtained the posterior density shown in Fig. 3g, based on which the compaction and persistence lengths are most likely to fall within the following ranges: *C* = 61 ± 4 bp/nm, *P* = 88 ± 4 nm (Fig. 3h, i). More conservative estimations were obtained when taking into consideration the discrepancy between the Hi-C and the imaging data, which our method does not account for, and using parameter ranges that span the above confidence intervals determined from both datasets separately. This led to an increase of the range for the persistence length to *P* = 52–85 nm, while that for the compaction remained similar at *C* = 53–65 bp/nm. These numbers provide new constraints on the average properties of chromatin in yeast and constitute a main result of this study (see “Discussion”). We note that in contrast to the compaction and rigidity parameters *C* and *P*, the diameter of the chromatin fiber, *W*, was not strongly constrained by the data (Additional file 1: Figure S5).

### Improved predictions of relative and nuclear locus positions

In addition to constraining chromatin parameters, our analysis yielded a model of the yeast nucleus that matches experimental data significantly better than our previous model [35]. To assess this, we chose the core model with the parameters closest to MAP estimates obtained from the combined data, namely the model with *C* = 50 bp/nm and *P* = 69 nm (Table 1, “best model”). With this model, we obtained a much better agreement between predicted and observed distances for 62 pairs of telomeres, as reflected by a Pearson correlation of *r* = 0.75 and an RMS error of 86 nm, compared to *r* = 0.64 and 173 nm, respectively, with the previous model [35] (Additional file 1: Figure S6). Furthermore, our improved model also faithfully recapitulated a new set of 55 distance measurements, including 12 new measurements between pairs of loci along the right arm of chromosome 4 (Fig. 4a). Interestingly, for a given genomic separation between the loci, our model predicted consistently larger distances for the pericentromeric region than for the internal region and this was indeed observed experimentally (Fig. 4b). This differential stretching of the pericentromeric chromosome region can be attributed in part to repulsion between the 32 chromosome arms, which are all confined by their centromere to the vicinity of the SPB. Indeed, in simulations of chromosome 4 where all other chromosomes were removed, the difference between pericentromeric and internal regions was reduced to less than half (Additional file 1: Figure S7). This behavior is qualitatively similar to the stretching of polymers in a polymer brush, where chains are grafted at one of their extremities on a common planar surface [59, 61].

We further compared the entire distributions of predicted distances to experimental measurements, for five intrachromosomal and four interchromosomal locus pairs [50] (Additional file 4). As shown in Additional file 1: Figure S8, the agreement between predictions and measurements was good or very good, even though only the median distances were used for parameter inference. This further highlights the model’s ability to accurately predict the entire statistical distributions of locus positions.

Our improved model also provides a better fit to the nuclear territories occupied by individual loci, as measured by the median angle between the locus, the nuclear center, and the nucleolar centroids [31, 50]. In our previous model, the predicted angles correlated well with the measured angles (Pearson’s *r* = 0.87), but were systematically underestimated by ~18° [35]. With our new model, the shift reduced to –3° and the correlation slightly improved to *r* = 0.92 with a RMS error of 8.6° (Fig. 4c).

### Improved predictions of genome-wide contact maps

We also compared the predicted genome-wide contact frequency maps to the two Hi-C datasets used for parameter inference above [29, 30] (Fig. 4d, e, Additional file 1: Figure S9a–c). When the contacts were binned at the highest resolution of 5 Kb, our improved model achieved a correlation of r = 0.65 or r = 0.85, with these data [29, 30] (Additional file 1: Figure S9f). This exceeded the correlation between the two Hi-C datasets themselves (r = 0.6) (Additional file 1: Figure S9f). The correlation of predicted contacts with either Hi-C dataset further improved when increasing the genomic bin, and exceeded r = 0.9 for bins of 70 Kb (Additional file 1: Figure S9f). To better assess our model’s predictive power, we further tested it against two additional genome-wide contact maps: a distinct Hi-C study by Belton et al. [62] (Fig. 4f), and a dataset obtained using Micro-C XL, a recently described variation of the Hi-C technique that uses alternative cross-linking agents instead of formaldehyde and DNA digestion by micrococcal nuclease instead of restriction enzymes [63] (Additional file 1: Figure S9e). Correlations between model and data were even higher for the Belton et al. Hi-C data and were only slightly lower for the Micro-C XL data (Additional file 1: Figure S9f). Since these two datasets were not used for parameter inference, this comparison further demonstrates the predictive power of our model.

The predicted mean contact frequencies among the 16 chromosomes also agreed very well with measurements from the three Hi-C data (Additional file 1: Figure S10a–d, f–h). We also analyzed the intra-chromosomal contact frequencies as function of genomic distance. We separately considered the genome-wide average or an average restricted to contacts involving a centromere (blue and green curves in Additional file 1: Figure S11, respectively). The measured contact frequencies decay faster in the centromeric region than elsewhere in the genome and this was also predicted by the simulation (Additional file 1: Figure S11). This effect is consistent with the peri-centromeric stretching observed on intrachromosomal distances above (Fig. 4b). The model’s agreement with the MicroC-XL data was also significant, though considerably less good (Additional file 1: Figure S10e, i), because this protocol appears to overestimate interchromosomal and long-range intrachromosomal contacts relative to Hi-C, perhaps due to an excess of random ligations (Additional file 1: Figure S10j, S11).

### Model recapitulates subdiffusive chromatin dynamics for multiple loci and time scales

Finally, although the model parameters were entirely inferred from static data—i.e. measurements from fixed cells or single snapshots of live cells—we decided to test our model’s ability to predict chromatin dynamics. We therefore compared the mean-squared displacements (MSD) of single loci, predicted by our simulation, to time-lapse microscopy data acquired in three recent studies [64–66] (Additional file 5) (Fig. 5). In order to map simulation time to physical time, we fitted a single scaling parameter once for each of the three datasets [64–66]. Figure 5a shows the predicted and measured MSD between 1 s and 150 s or between 1 s and 10 s for seven different loci on the right arm of chromosome 4 (4R) and for the MAT locus on chromosome 3 [65, 66]. The distances of loci to the centromere were in the range of 12–985 Kb, spanning over 90% of the 1050 Kb long chromosome arm 4R except for the immediate vicinity of the centromere and telomere. As apparent from Fig. 5a, the model approximately reproduced the measured MSD and recapitulated the observed ordering, i.e. the fact that loci at larger genomic distance from the centromere moved over longer distances during the same time interval. Figure 5b–e shows measured MSD for four loci on four different chromosomes (including the rDNA-carrying chromosome 12, whose spatial configuration is quite different from the other chromosomes [64]) over time intervals in the range of 16 ms to 100 s. As previously pointed out, the MSD obeyed a subdiffusive power law with an exponent ~0.5, roughly consistent with the Rouse polymer model [18, 37, 41, 44, 64]. The model reproduced this behavior relatively well over almost four orders of magnitude of time. Discrepancies between predictions and measurements were similar to or smaller than internal discrepancies within the experimental datasets (Fig. 5b–e). These results confirm the ability of our model to correctly predict the dynamic behavior of chromatin, even though its structural parameters were inferred exclusively from static data.

## Discussion

In this paper, we described a new approach to infer mechanical parameters of chromatin using a whole nucleus simulation of chromosomes. Compared to methods that estimate hundreds or thousands (or more) parameters to reproduce Hi-C contact data or other genome-wide data [17, 24, 25, 67, 68], our model assumes only a small number of structural parameters, giving it high predictive power. Our Bayesian formulation and sampling approach allow to determine the region of parameter space consistent with the data and to assign probabilities to parameter values. This approach thereby provides information about the uncertainties associated to the inferred parameters for each dataset or, equivalently, about how strongly the data constrain the parameters. We applied our method to a large set of experimental data on yeast nuclear architecture. This analysis led to two main outcomes: new constraints on yeast chromatin and a better model of yeast nuclear organization.

First, we derived new constraints on yeast chromatin compaction and rigidity, with implications for chromatin structure. Although several previous studies have attempted to determine these parameters from imaging or Hi-C data [5, 39–42], they typically used either a single experimental dataset (e.g. only distances between loci, which are insufficient to unambiguously determine compaction and rigidity simultaneously, as shown in Figs. 2a and 3a) and/or used simpler polymer models that ignored important aspects of nuclear organization, such as tethering of centromeres and telomeres, or entropic repulsion between chromosomes. Accordingly, estimates of compaction *C* and persistence length *P* from previous studies spanned a wide range: 30–150 bp/nm and <30–200 nm, respectively. By contrast, our analysis is based on a much more varied and complete dataset compiled from multiple independent studies, which include distances between loci in *cis* and *trans*, nuclear locus territories, and two Hi-C datasets. We analyzed these data using a chromosome model that accounts for nuclear confinement, steric hindrance among chromosomes, tethering at centromere and telomeres, and the nucleolar compartment. This more elaborate model accounts for non-trivial features of chromosome organization, such as stretching of peri-centromeric chromatin (Fig. 4b). Most importantly, our analysis supports a relatively narrow range of values for chromatin compaction *C* (53–65 bp/nm) and significantly constrains the bending persistence length *P* (52–85 nm) in yeast chromatin.

Given a nucleosome repeat length of 167 bp in yeast [69], this estimated compaction corresponds to approximately four nucleosomes per 11 nm turn of the DNA double helix. This has implications for the possible structures of the chromatin fiber and can be confronted to two textbook structures: the 30 nm fiber, in which the ≈ 11 nm diameter nucleosomes are tightly packed together in a compact 3D structure of ~30 nm diameter, and the beads-on-a-string structure, where consecutive nucleosomes are arranged in a much looser ~10 nm diameter fiber. The classical 30 nm fiber has a compaction of ~11 nucleosomes per 11 nm, much larger than our estimate above [70]. Because of its short nucleosomal repeat length, the yeast chromatin fiber can be expected to adopt a somewhat looser ~20 nm (rather than 30 nm) diameter structure, with a compaction of approximately six nucleosomes per 11 nm [69, 70]. This is, however, still too compact to agree with our estimates. Thus, our results cast doubt on the existence of a compact 20–30 nm fiber throughout the yeast genome. They complement previous studies questioning the existence of 30 nm fibers in other organisms [4, 71, 72]. On the other hand, a stretched 10 nm fiber, with a compaction of approximately one nucleosome per 11 nm, is insufficiently compact to fit our estimates [73]. Thus, the possibility arises that the chromatin fiber has a structure quite different from both textbook structures.

As a caveat, since our model rests on the assumption of a homogeneous chromatin fiber throughout the genome (except for the rDNA region), our estimates only pertain to the average properties of the fiber. The chromatin fiber is potentially heterogeneous and may consist of alternating stretches with different compaction and rigidity. Hi-C data on the human genome, for example, indicate that chromosomes are partitioned into “close” and “open” regions of chromatin on the order of 10 Mb, most readily identified as blocks of negative and positive values in contact correlation matrices (Additional file 1: Figure S12a) [15]. In contrast, correlation matrices of budding yeast Hi-C data [62] at genomic resolutions of 10 Kb remain quite homogeneous at high positive correlations, except in the pericentromeric region where correlations are strongly negative (Additional file 1: Figure S12b). Although this pericentromeric pattern might first be interpreted as a signature of chromatin heterogeneity, it is in fact recapitulated by our homogeneous fiber model (Additional file 1: Figure S12c), confirming that homogeneity is a valid assumption at this genomic scale. Nevertheless, heterogeneities at smaller scales, such as rapid alternations between compact and less compact chromatin might in principle lead to detectable structures in the contact matrix (Additional file 1: Figure S13a, c). Such signatures are hard to identify in the Hi-C data [62] (Additional file 1: Figure S13d), although they may be present in the raw Micro-C XL data (Additional file 1: Figure S13e). However, reliably distinguishing these signatures from potential biases [54, 74, 75] is challenging, and none of the observables used for parameter inference in our study is sensitive to such small-scale heterogeneities (Additional file 1: Figure S14). Although large-scale heterogeneities are not supported by the Hi-C data, we therefore cannot rule out small scale heterogeneities, e.g. rapidly alternating stretches of compact 20–30 nm fibers and loose 10 nm fibers. For such a heterogeneous structure, assuming the above compaction values, we can estimate that roughly half of the linear length of the chromatin fiber (ignoring the non-rDNA yeast genome) would be structured as a compact 20–30 nm fiber and the other half as a 10 nm beads-on-a-string fiber. Accounting for the different compactions, these proportions correspond to 86% and 14% of the genome, respectively. Our modeling approach cannot currently map these potential regions to the genome. Extending our method to heterogeneous fibers at small (<5 Kb) genomic scales would imply estimating thousands of parameters at the risk of overfitting and loss of predictive power. Nonetheless, future heterogeneous models that avoid overfitting might leverage state-of-the art genomic contact data and high resolution imaging data to shed more light on potential structural variations of the chromatin fiber [12, 24, 42, 60, 76].

In addition to new insights into average yeast chromatin structure, our analysis yielded a set of models that provide a better explanation of experimental data than our previous model [35]. In particular, for parameters that maximize the posterior probability, the model agrees well with measured distances between loci (means and distributions), intranuclear gene territory positions, and several aspects of Hi-C data, including Hi-C data that were not used for parameter estimation. Moreover, unlike Monte-Carlo simulations or structural ensembles obtained by optimization [24, 36], our model can predict the dynamic behavior of chromatin, in good agreement with observations. Based on these results, it is now possible to make accurate predictions of absolute and relative locus positions, movements and contact frequencies throughout the yeast genome. This in turn has implications for a quantitative understanding of functional processes such as DNA repair by homologous recombination and mating type switching in yeast [37, 38, 77, 78].

## Conclusion

In summary, our work provides new insights into yeast chromatin fiber structure, and proposes a substantially improved predictive model of yeast nuclear architecture and dynamics, both of which will help to advance our understanding of structure-function relations in the nucleus. The computational analysis method proposed here should also be applicable to characterizing chromatin structure and chromosome organization in many other organisms.

## Additional files

Additional file 1: Supplementary information. Includes: Supplementary **Figure S1–S19**; Supplementary Methods A–F; Supplementary References. (PDF 19000 kb)

Additional file 2: Table listing all 144 models used for inference and their parameter values. (XLSX 40 kb)

Additional file 3: Table listing all image-based distance measurements used for parameter inference. (XLSX 77 kb)

Additional file 4: Entire set of measured distances between nine pairs of loci used to analyze distance distributions. (XLSX 160 kb)

Additional file 5: Mean square displacements measured on multiple chromatin loci in independent studies. (XLSX 79 kb)

Additional file 6: Zipped archive containing the scripts to run the best model simulation. (ZIP 241 kb)
