# Supplementary material for: Inferring the physical properties of yeast chromatin through Bayesian analysis of whole nucleus simulations
Source: Genome Biol. 2017 May 3;18:81. doi: 10.1186/s13059-017-1199-x (PMC5414205; doi:10.1186/s13059-017-1199-x)
Supplement: Supplementary file 1 — Supplementary information. Includes: Supplementary Figure S1–S19; Supplementary Methods A–F; Supplementary References. (PDF 19000 kb) [file 13059_2017_1199_MOESM1_ESM.pdf]

Supplementary Information

“Inferring the physical properties of yeast chromatin through Bayesian analysis of whole nucleus simulations”

Jean-Michel Arbona, Sébastien Herbert, Emmanuelle Fabre, Christophe Zimmer

Supplementary Figures:

Figure S1: Initialization and sampling of the simulation 2
Figure S2: Similar predictions from replicate simulations 3
Figure S3: Parameters of the simulations used for validation 4
Figure S4: Robustness of parameter inference to parameter mismatch 5
Figure S5: Weak effect of chromatin width on parameter estimation 6
Figure S6: Improved prediction of distances between telomeres 7
Figure S7: Pericentromeric chromatin stretching is due to chromosome crowding 8
Figure S8: Predicted vs measured distance distributions between pairs of loci. 9
Figure S9: Predicted vs measured genome-wide contact frequencies 11
Figure S10: Predicted vs measured contact frequencies between chromosomes 12
Figure S11: Predicted vs measured intrachromosomal contact frequencies 13
Figure S12: Contact correlation maps for human and yeast chromosomes 14
Figure S13: Effect of fiber heterogeneity on contact frequency maps 15
Figure S14: Predictions of heterogeneous vs homogeneous fiber models 16
Figure S15: FENE and bending potentials 18
Figure S16: Repulsion between monomers. 19
Figure S17: Potentials for nuclear confinement and telomeric tethering. 20
Figure S18: Centromeric tethering potential 21
Figure S19: Effect of capture radius on predicted contact probabilities 24

Supplementary Methods:

A. Langevin dynamics simulations 17
B. Predicting observables from simulations 23
C. Bayesian inference and priors 26
D. Generation of synthetic validation data 30
E. Genome-wide contact frequency data 31
F. Intrachromosomal distances on chromosome 4 32

Supplementary references: 33

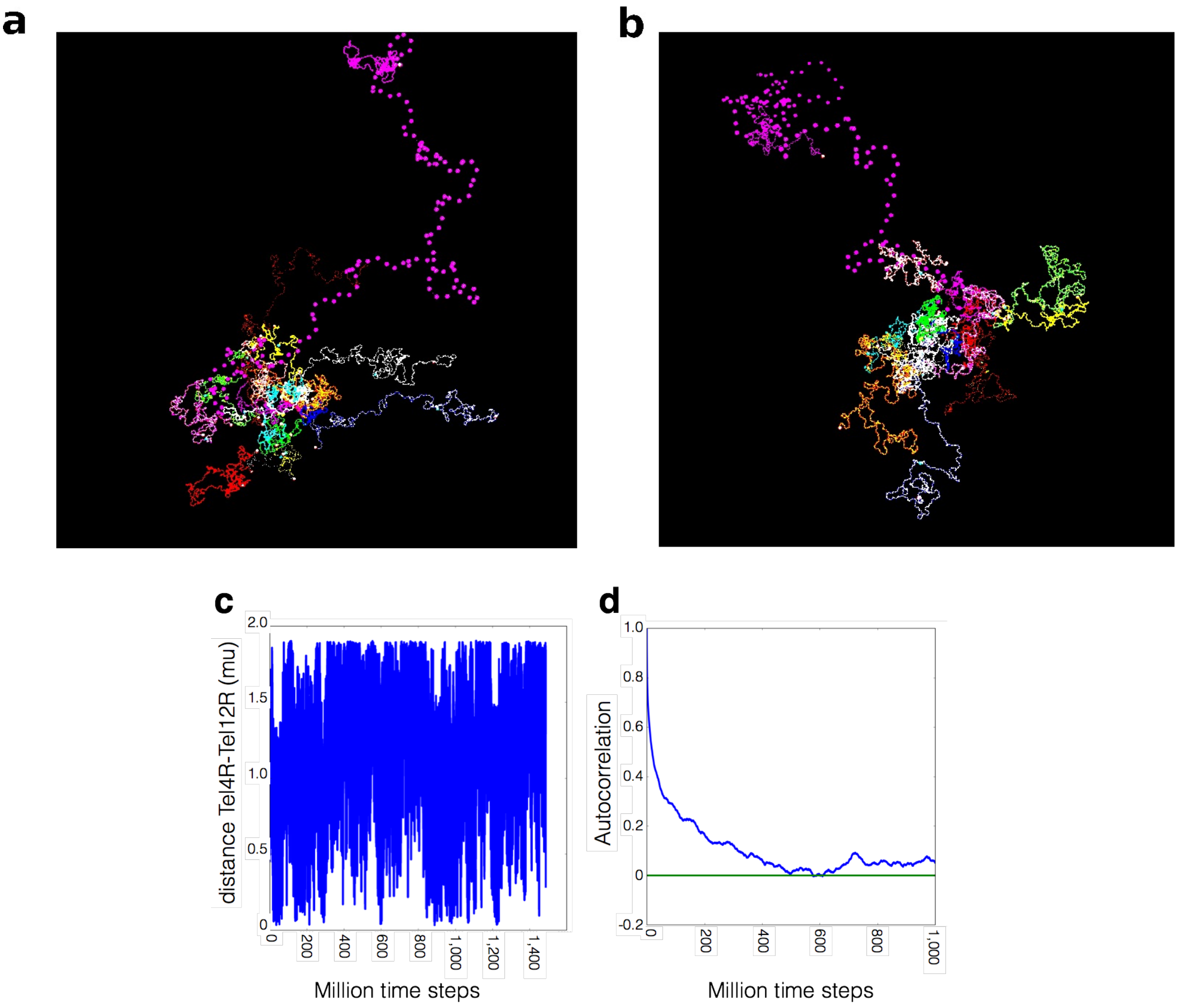

Figure S1: Initialization and sampling of the simulation

a,b) Each simulation run is initialized by a random configuration of chromosomes as illustrated. Two initializations are shown, for the same model parameters. Beads belonging to the same chromosome have the same color. Pink beads represent telomeres, and the bigger violet beads are monomers of the rDNA locus. **c**) Distance between the beads corresponding to the telomeres of chromosome arms 4R and 12R as function of simulation time steps. **d**) Autocorrelation function of the distances shown in **c**. Distances become effectively decorrelated after ~$3x10^7$ time steps. Because chromosome arms 4R and 12R are the longest in the yeast genome, they are expected to dominate the decorrelation time. Results shown here are for the simulation containing the largest number of monomers (n=16,202), for which the decorrelation time is expected to be largest. The simulation used the following parameters: persistence length P = 55 nm, C = 25 bp/nm, W = 30 nm, L = 400 nm. The entire simulation was run for 10^{10} time steps. After a burn-in period of 10^8 time steps, we sampled the simulation trajectories every 10,000 time steps and pooled the samples from 6 independent replicate simulations (2-6 replicates were used for each model depending on the parameter values, see **Additional File 2**).

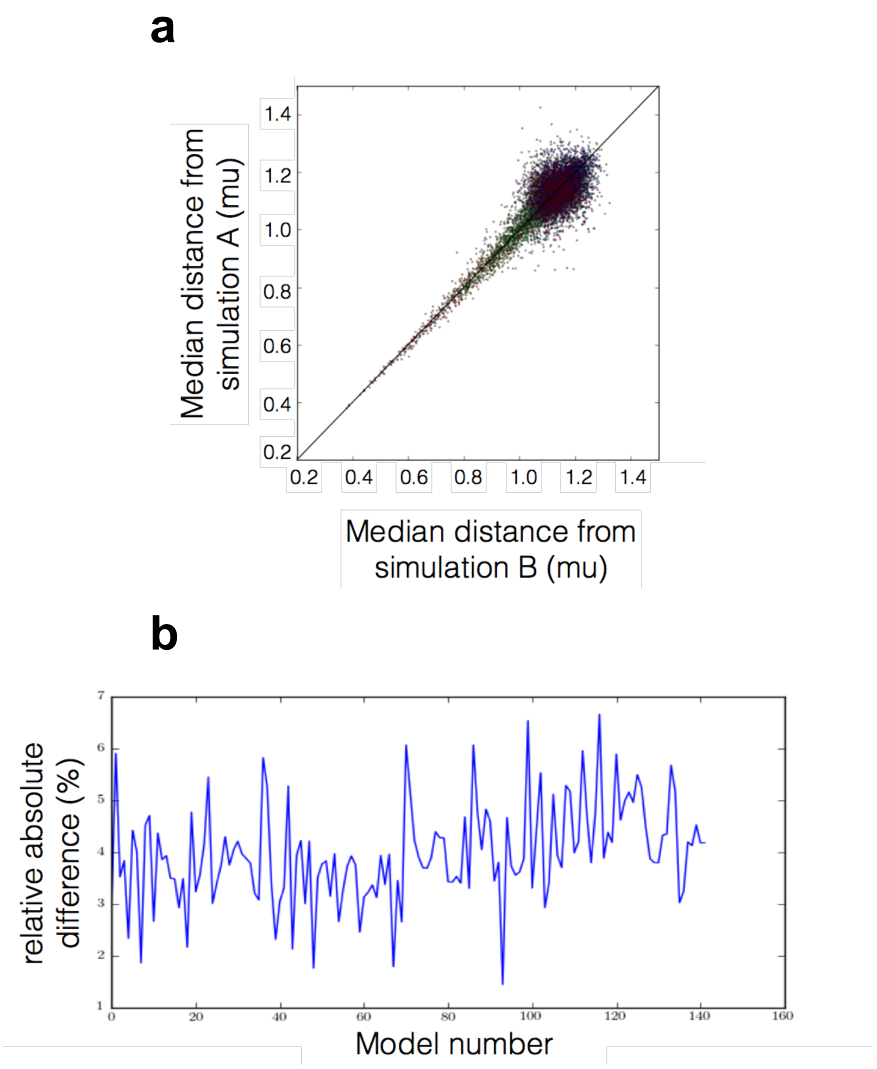

Figure S2: Similar predictions from replicate simulations

a) for each of the 144 core models (**Table 1, Additional File 2**), this plot shows the median telomere-telomere distances for 62 pairs of telomeres[1] (observable O1, **Table 2** and **Additional File 3**) as predicted by two independent simulation runs A and B for the same parameter values Π_i (or the averages from two groups of independent simulations when more than 2 replicates were available). Each dot corresponds to a single parameter value $\Pi_i, i = 1..144$, and a single pair of telomeres. Each color corresponds to a single pair of telomeres. **b**) The relative mean absolute difference (in %) between median distances predicted by two replicate simulations, for each value of the parameter Π_i (as indicated by simulation number *i*; see **Additional File 2**).

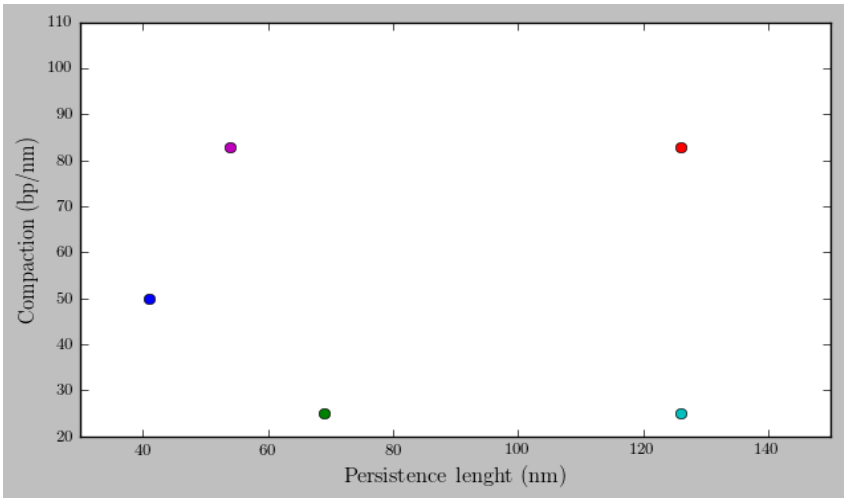

Figure S3: Parameters of the simulations used for validation

To validate the parameter inference method on a known ground truth, we generated synthetic data from our simulation. We did this for five different values of the chromatin compaction *C* and persistence length *P* as indicated by the five dots: (P_0, C_0)= (41 nm, 50 bp/nm); (69 nm, 25 bp/nm); (126 nm, 83 bp/nm); (126 nm, 25 bp/nm), and (54 nm, 83 bp/nm). The chromatin width and microtubule length were fixed to W = 45 nm and L = 300 nm in all five cases.

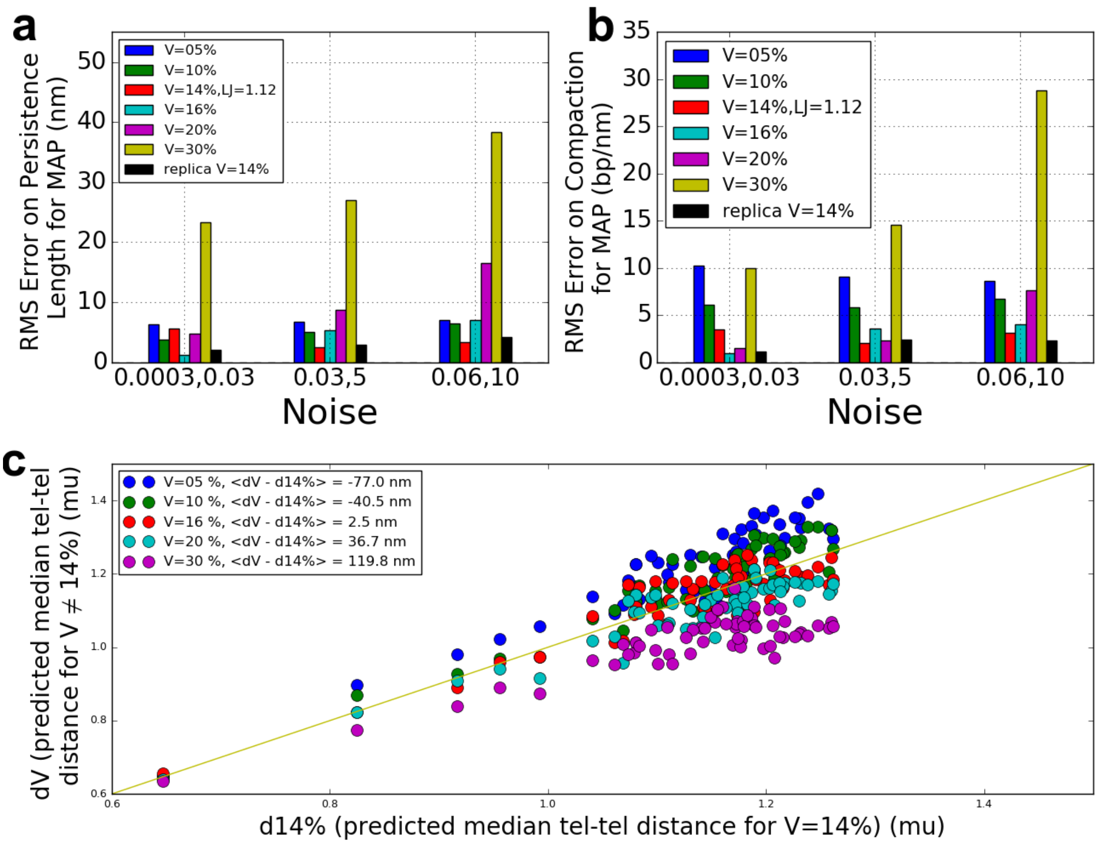

Figure S4: Robustness of parameter inference to parameter mismatch

Results of a robustness analysis, where the inference method was applied to synthetic data generated by a simulation with parameter values outside the range spanned by the core simulations (see **Fig. 1a**, **Table 1** and **Additional File 2**). Specifically, we changed W_{rDNA}, the diameter of the beads corresponding to the rDNA locus, such that the net volume occupied by these beads, *V*, varied from 5% to 30% of the nuclear volume, with intermediate values 10%, 16% and 20%. The total volume effectively occupied by these beads is roughly 2*V*. In all core simulations, we used *V* = 14% (**Additional File 2**). **a**) Bars show the root mean squared (RMS) error of the chromatin persistence length *P* as determined from the maximum a posteriori (MAP) estimate (see **Fig. 1f,g**). Three different levels of noise were added to the simulated data (**Supplementary Methods** section D). Each color corresponds to a different volume *V*, as indicated in the legend. The black bar corresponds to an independent replicate of the core simulation with *V* =14%. The red bar corresponds a simulation with *V* =14% but using a cutoff distance of 1.12 for the Lennard-Jones potential instead of 1.15 (see **Supplementary Methods** section A.3). **b**) same as panel a, for the chromatin compaction *C*. **c**) Predicted median distances between 62 pairs of telomeres (observable O1, **Table 2** and **Additional File 3**) for different volumes *V* compared to the reference simulation with *V* = 14 %. The average difference between the distances predicted for *V*≠14% and *V*=14% is indicated in the legend.

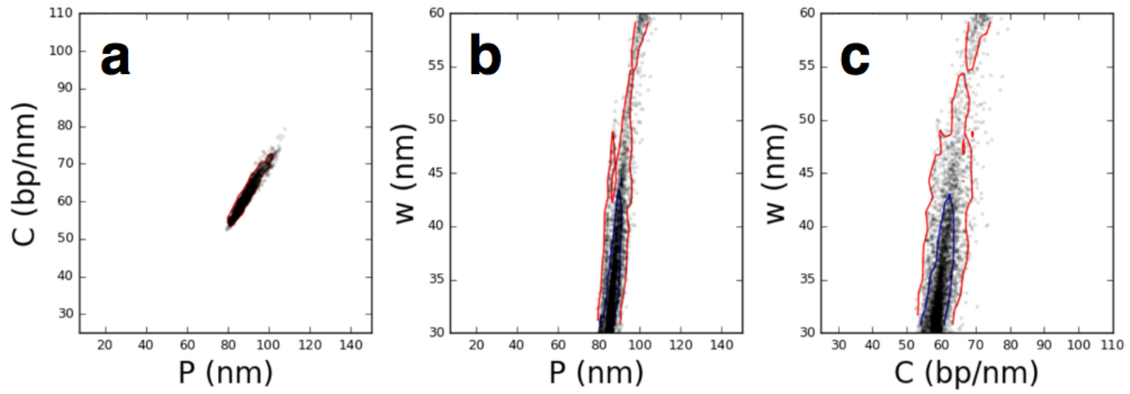

Figure S5: Weak effect of chromatin width on parameter estimation

These plots show the joint posterior probability density for the three pairs of parameters (*C,P*), (*W,P*) and (*W,C*) among the chromatin compaction *C,* the chromatin persistence length *P*, and the chromatin fiber width *W,* as determined from the entire experimental data set combined (observables O1-O9, see **Table 2** and **Additional File 3**). Panel a is identical to **Fig. 3g**. The data constrain both *C* and *P* to a relatively narrow range (**a**). By contrast, it is apparent from the almost vertical probability densities in **b** and **c** that the data do not strongly constrain *W.* Thus, the estimation of *P* and *C* is not sensitive to the exact values of *W* within this range.

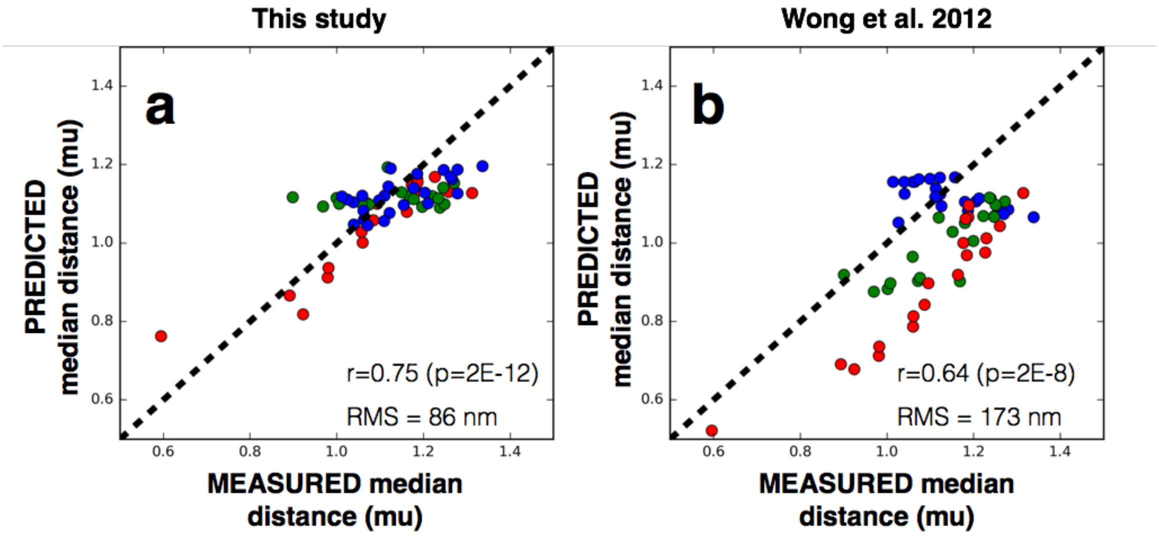

Figure S6: Improved prediction of distances between telomeres

Both panels show the predicted vs measured median distances between 62 pairs of telomeres (observable O1, **Table 2** and **Additional File 3**). Blue dots are for telomere pairs involving telomere 4R, red dots are for pairs involving telomere 6R, green dots are for pairs involving telomere 10R. **a**) Predictions are from our "best model" (**Table 1**). **b**) Predictions from our previous study by Wong et al. [2]. The Pearson correlation coefficient between predicted and measured distances and the root mean square (RMS) error are indicated. The model predictions are in markedly better agreement with the measurements in panel **a**. Note in particular that for distances between telomere 4R and other telomeres (blue dots), predictions negatively correlated with the measurements in Wong et al. [2] (panel **b**), indicating systematic discrepancies. Such negative correlations are no longer apparent in the new model (panel **a**).

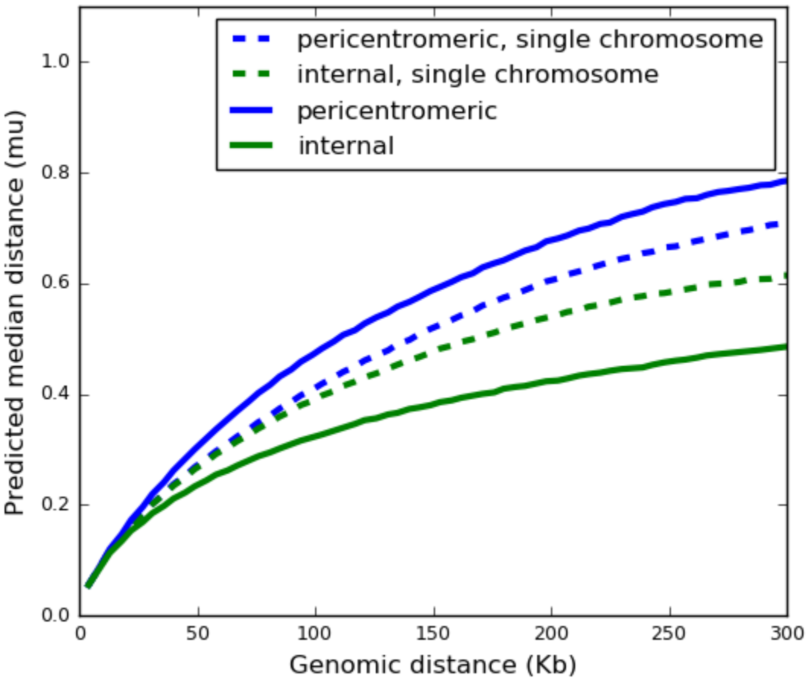

Figure S7: Pericentromeric chromatin stretching is due to chromosome crowding

The predicted median spatial distance between two loci on the right arm of chromosome 4 is plotted as function of the genomic distance *s* between the loci, both for the pericentromeric (blue) and internal region of the chromosome (green). For the blue curves, one of the two loci is at 4 Kb from the centromere; for the green curves, one of the loci is at 404 Kb from the centromere and 678 Kb from the telomere. Solid curves are from a core simulation, which accounts for steric and topological constraints among chromosomes (model #17 in **Additional File 2**). Dashed curves are from a simulation with the same parameters, but in which all chromosomes except chromosome 4 were removed. In both simulations, spatial distances increase with genomic separation *s*, but for a given genomic distance *s*, the spatial distance between loci is larger near the centromere than in the internal region of the chromosome arm, reflecting pericentromeric stretching of the chromatin fiber (and conversely “shrinking” of the internal chromosome region). This difference between pericentromeric and internal regions is strongly reduced when the other chromosomes are removed from the simulation, indicating that it arises largely from steric or topological constraints exerted by the other chromosomes.

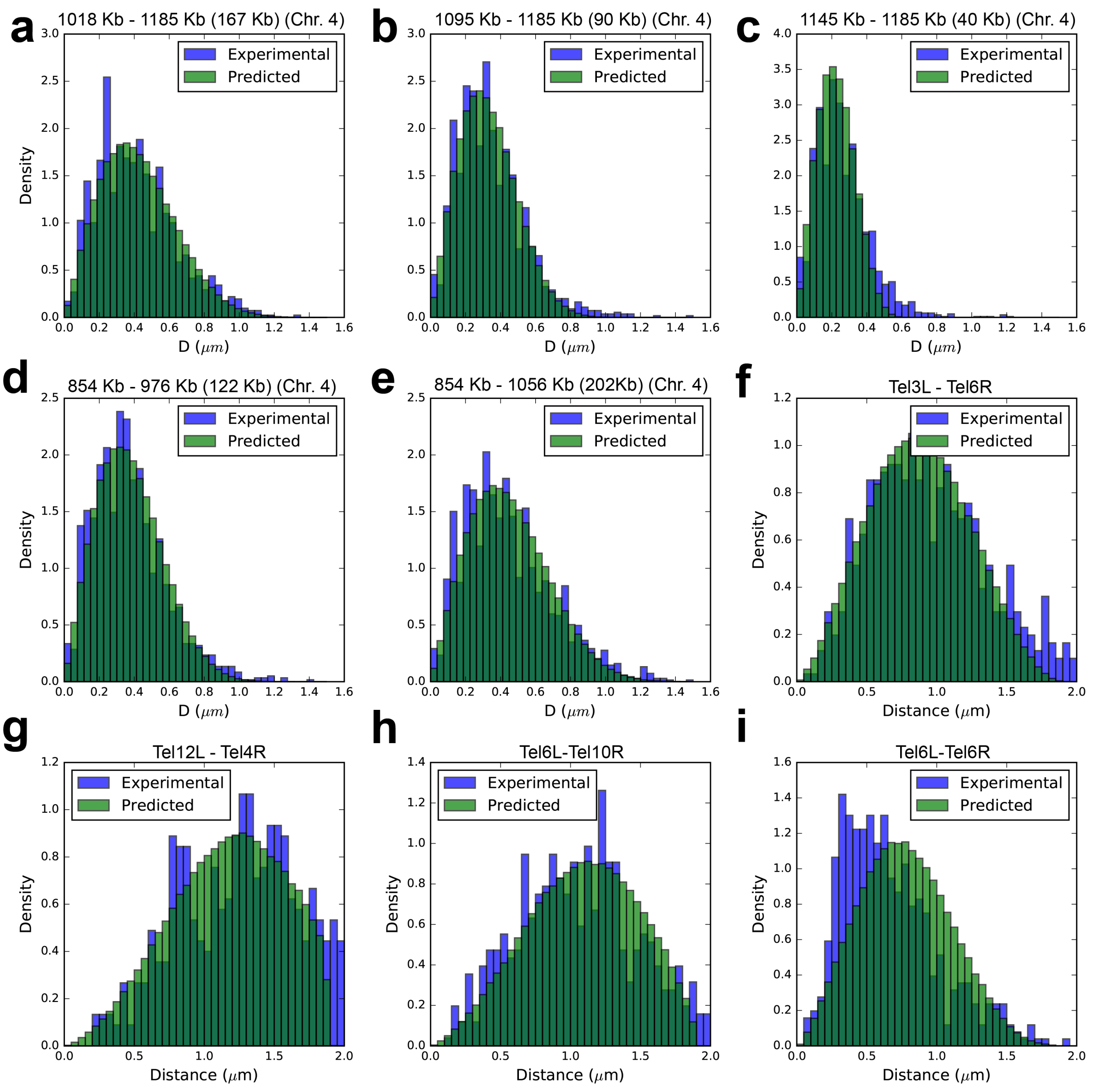

Figure S8: Predicted vs measured distance distributions between pairs of loci.

Predicted (green) and measured (blue) distance distributions for nine pairs of chromatin loci. Experimental data are provided in **Additional File 4**. **a-e)** distances between pairs of loci on the right arm of chromosome 4. Panel titles indicate the genomic distance of the two loci to the centromere, with the genomic distance between them in parentheses. **f-I)** distances between pairs of telomeres. Panel titles indicate the chromosome arm of each telomere (e.g. Tel3L is the telomere on the left arm of chromosome 3).

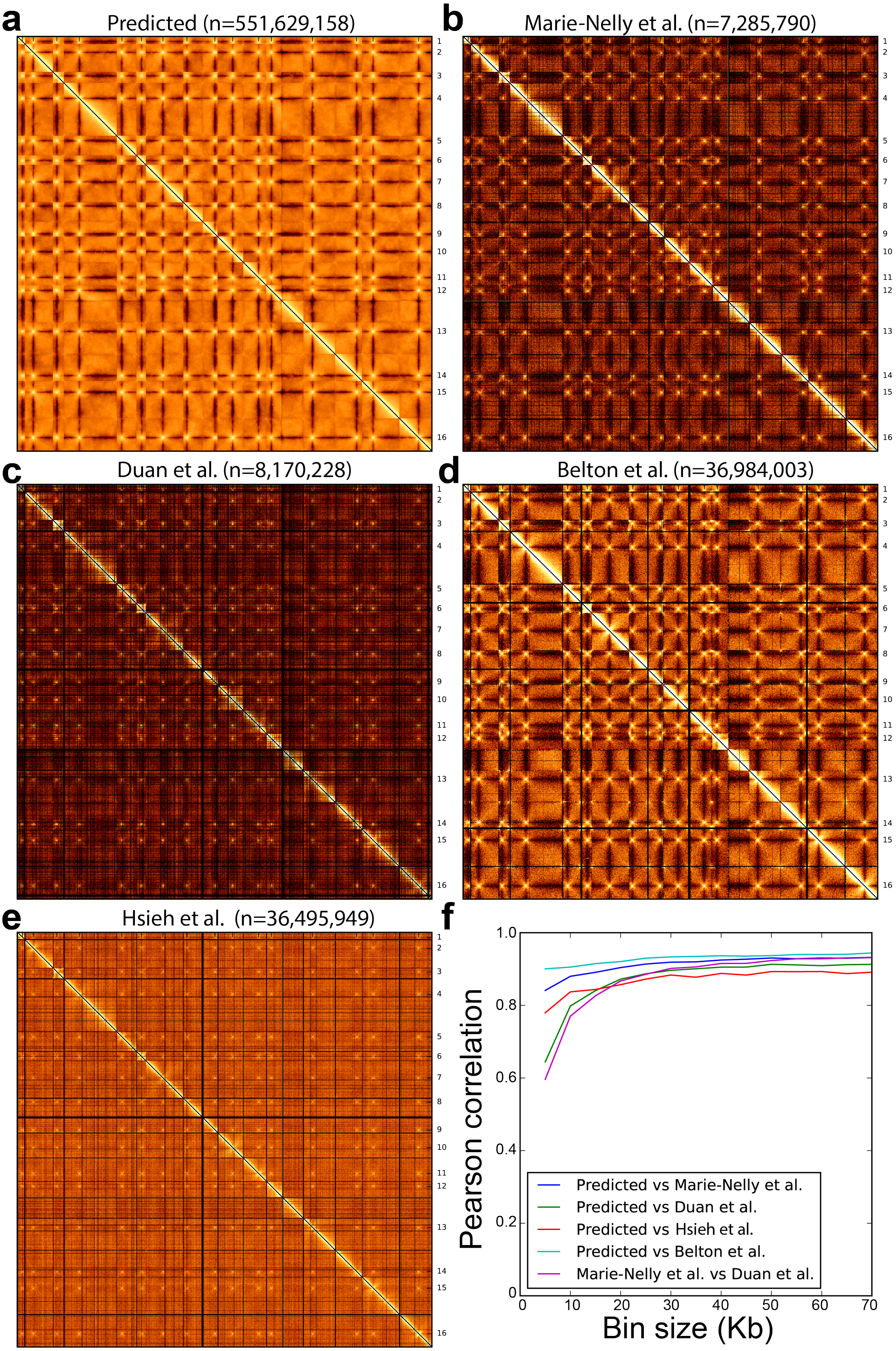
a
Predicted (n=551,629,158)
b
Marie-Nelly et al. (n=7,285,790)
c
Duan et al. (n=8,170,228)
d
Belton et al. (n=36,984,003)
e
Hsieh et al. (n=36,495,949)
f
Pearson correlation
Bin size (Kb)
Predicted vs Marie-Nelly et al.
Predicted vs Duan et al.
Predicted vs Hsieh et al.
Predicted vs Belton et al.
Marie-Nelly et al. vs Duan et al.

Figure S9: Predicted vs measured genome-wide contact frequencies

Predicted (**a**) and experimentally measured (**b-f**) genome-wide contact frequency matrices. The predicted contact frequencies are from our "best model" (**Table 1**). The measured contact frequencies were obtained from three independent Hi-C studies by Marie-Nelly et al. [3] (**b**), Duan et al. [4] (**c**), Belton et al. [5] (**d**) and one Micro-C XL experiment by Hsieh et al. [6] (**e**). For all matrices the bin size is 5 Kb. Contact frequencies are displayed as heat maps, with bright pixels indicating high frequencies and dark pixels low frequencies. A logarithmic scaling is applied to reveal lower frequency contact patterns. **f**) Pearson correlation coefficient between predicted and measured chromatin contact frequencies, as function of genomic bin size from 5 Kb to 70 Kb. Blue: correlation between model prediction and Hi-C data from Marie-Nelly et al.[3] (O9, see **Table 1**). Green: correlation between model prediction and Hi-C data from Duan et al.[4] (O8, see **Table 1**). Red: correlation between model predictions and Micro-C XL data from Hsieh et al. [6]. Cyan: correlation between model predictions and Hi-C data from Belton et al. [5]. Violet: correlation between Hi-C data from Marie-Nelly et al.[3] and Duan et al.[4].

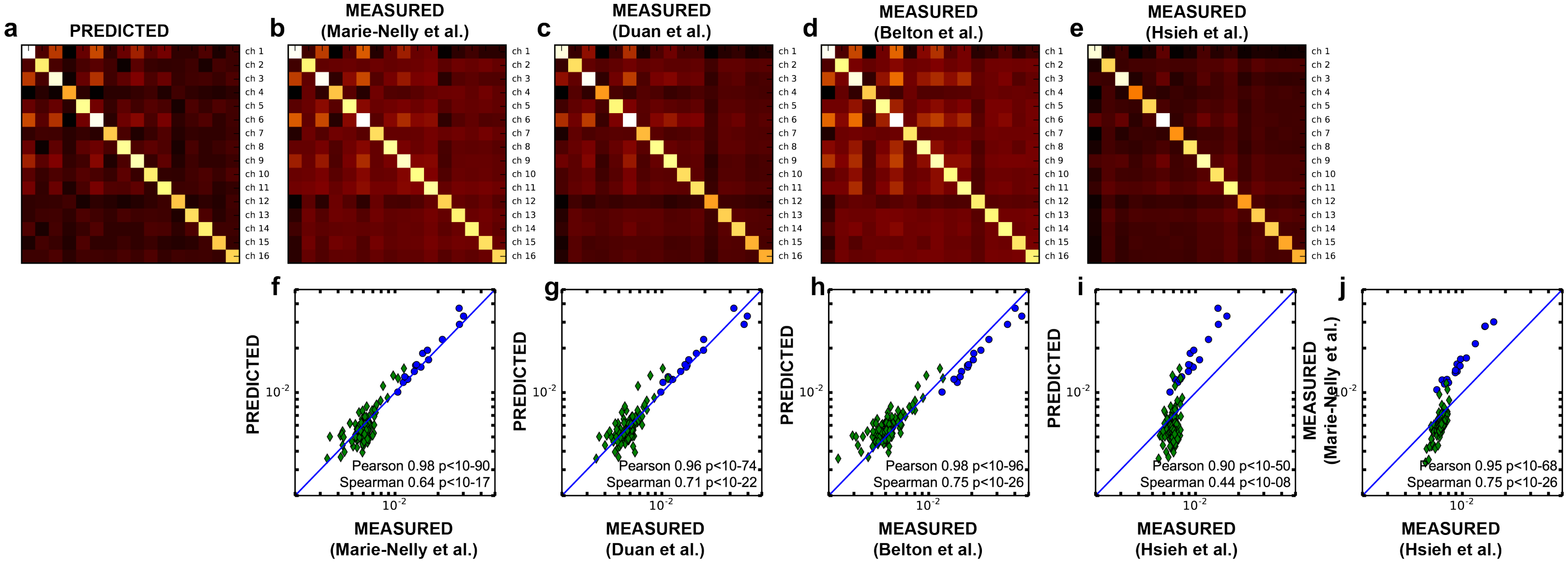

Figure S10: Predicted vs measured contact frequencies between chromosomes

a-e) Predicted (a) and measured (b-e) contact frequencies between the 16 chromosomes, displayed as heat maps with bright colors indicating high frequencies, and dark colors indicating low frequencies. The contact frequencies are normalized by the product of genomic lengths of the chromosomes to remove trivial variations [2]. Predicted contact frequencies (**a**) are from our "best model" (**Table 1**). Measured contact frequencies are from Hi-C data by Marie-Nelly et al.[3] (**b**), Hi-C data by Duan et al.[4] (**c**), Hi-C data by Belton et al. [5] (**d**) and Micro-C XL data by Hsieh et al. [6] (**e**). **f-i**) Scatter plots show the predicted vs measured contact frequencies from **a-e**. Blue circles correspond to intrachromosomal contacts, green diamonds to interchromosomal contacts. Pearson and Spearman correlation coefficients are indicated along with the corresponding p-value.

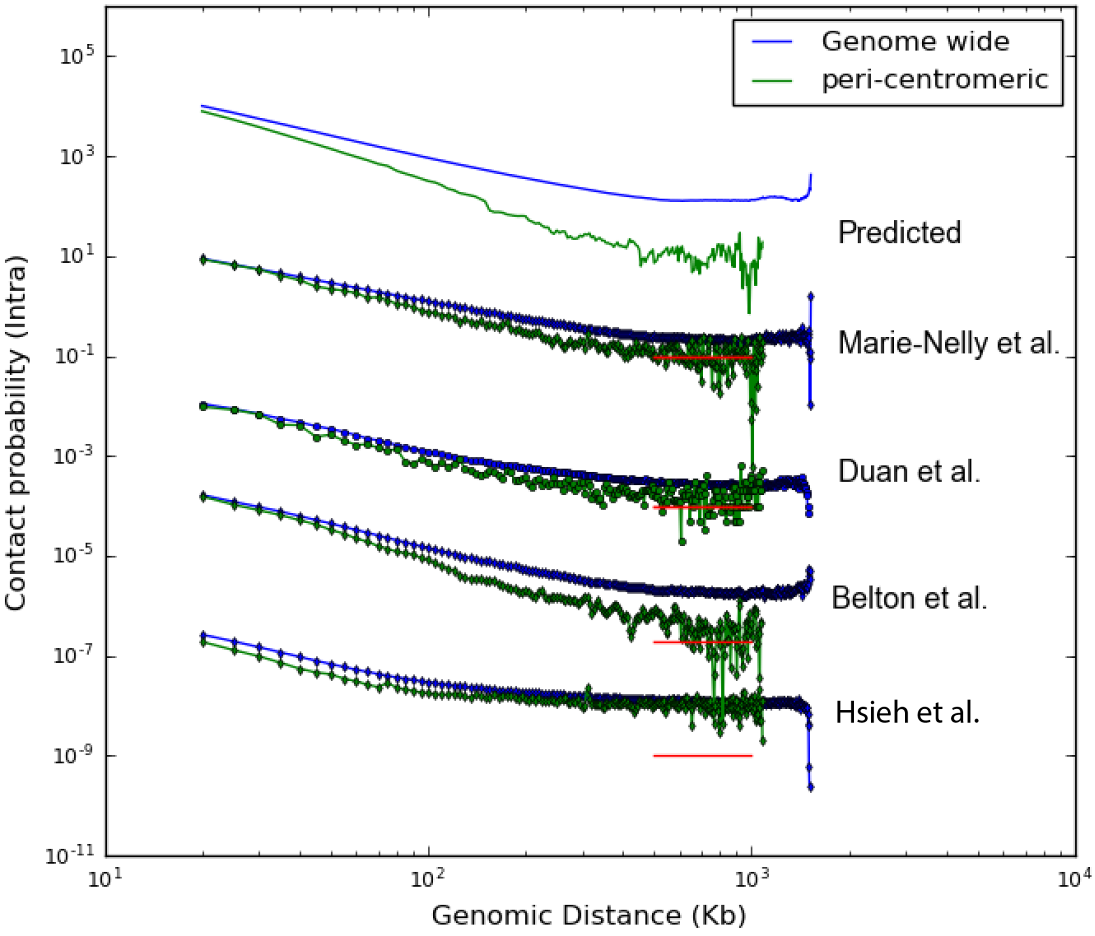

Figure S11: Predicted vs measured intrachromosomal contact frequencies

Intrachromosomal contact probabilities are plotted as function of genomic distance. Contact probabilities are scored using bins of 5 Kb. Blue curves are genome-wide averages. Green curves are restricted to contacts involving a centromere. From top to bottom: predictions from our “best model” (**Table 1**); Hi-C data from Marie-Nelly et al.[3] (09); Hi-C data from Duan et al.[4] (O8); Hi-C data from Belton et al. [5]; Micro-C XL data from Hsieh et al. [6]. The three pairs of curves were scaled differently to facilitate visualization. For the experimental data, red lines indicate the value corresponding to a single contact event.

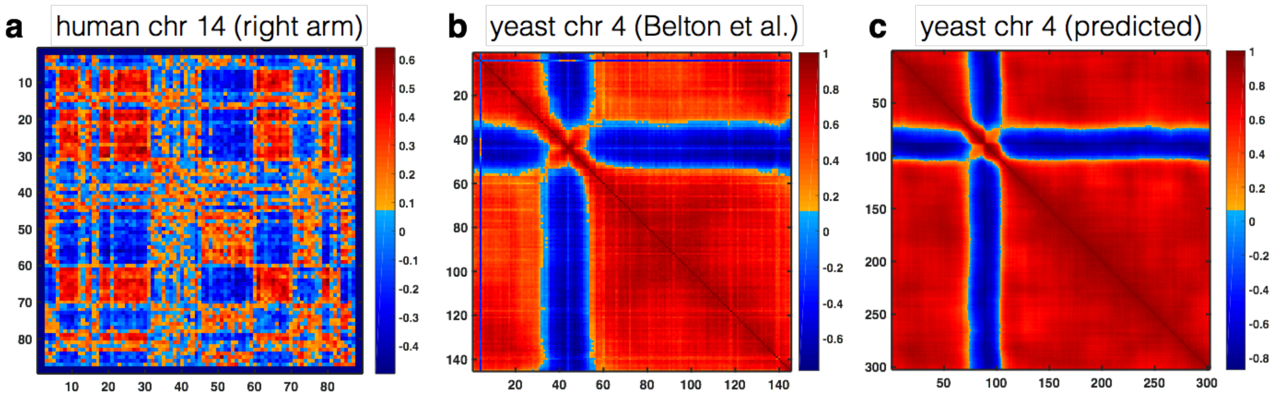

Figure S12: Contact correlation maps for human and yeast chromosomes

The three panels show correlation matrices calculated from intrachromosomal contact data obtained by Hi-C experiments (**a**,**b**) or predicted by our model (**c**). Correlation matrices were computed as first described in [7]: in order to remove the average effect of genomic distance on the contact frequencies, the raw contact frequency matrix was normalized by the average contact frequencies over all pairs of loci that share the same genomic distance; then the correlation matrix was computed such that each entry *(i,j)* is the Pearson correlation between rows *i* and *j* of the normalized contact frequency matrix. **a**) Contact correlation matrix for the right arm of human chromosome 14, with a bin size of 1 Mbp, as obtained from Hi-C data in Lieberman-Aiden et al. [7]. **b**) Contact correlation matrix for yeast chromosome 4, as obtained from Hi-C data in Belton et al. [5], with a bin size of 10 Kb. **c**) Contact correlation matrix for yeast chromosome 4 as predicted by our "best model", with a bin size of 5 Kb. While multiple large compartments of negative or positive correlations are clearly apparent in the Hi-C data of the human chromosome (**a**), this is not the case in Hi-C data of yeast, where correlations are high and homogeneous except for the pericentromeric region, which exhibits negative correlations (**b**). A very similar pattern is predicted by our model, which assumes an entirely homogeneous chromatin fiber (**c**). Therefore, reproducing the observed contact correlation pattern does not require a heterogeneous fiber model.

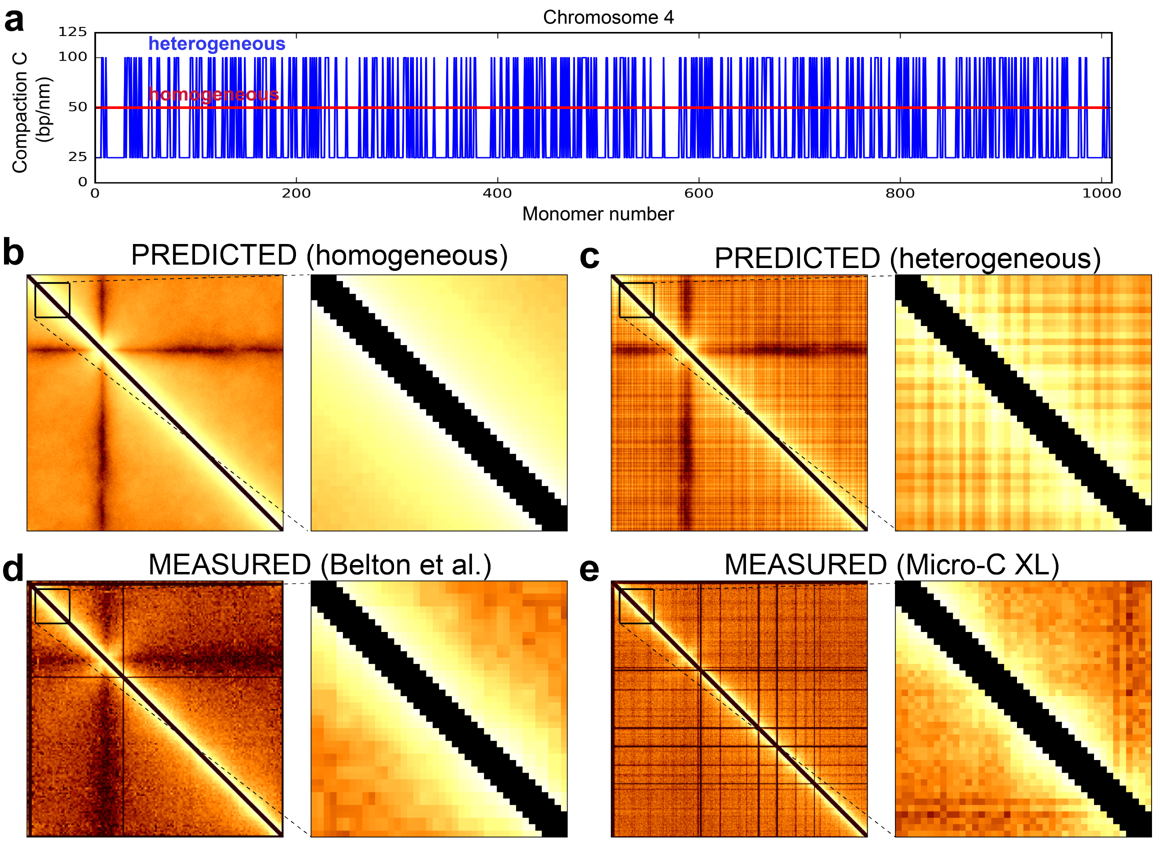

Figure S13: Effect of fiber heterogeneity on contact frequency maps

We examine a heterogeneous fiber model consisting of a mixture of monomers of high compaction (C_1 = 100 bp/nm) and monomers of low compaction (C_2 = 25 bp/nm) and compare it to our "best model", which assumes a homogeneous chromatin fiber with a constant compaction of C = 50 bp/nm. For each chromosome arm, the number of compact monomers was set to $N_1 = (C - C_2)/(C_1 - C_2)N$, where N is the total number of monomers in the homogeneous fiber model. A random subset of N_1 out of N monomers was assigned a high compaction C_1, while the remaining $N_2 = N - N_1$ monomers were assigned low compaction C_2. This ensures that the average compaction along each chromosome arm equals C, as in the homogeneous model. **a**) compaction as function of monomer index along chromosome 4 for the heterogeneous fiber (blue) and the homogeneous fiber (red). **b-e**) predicted (**b,c**) and measured (**d,e**) contact frequency maps for yeast chromosome 4, for a bin size of 5 Kb, together with a magnified view of a 200 Kb region on the diagonal. **b**) prediction from the homogeneous fiber model. **c**) prediction from the heterogeneous fiber model. Horizontal and vertical stripes result from variations in chromatin compaction along the chromosome. **d**) Hi-C contact data from Belton et al. [5]. **e**) Micro-C XL contact data from Hsieh et al. [6].

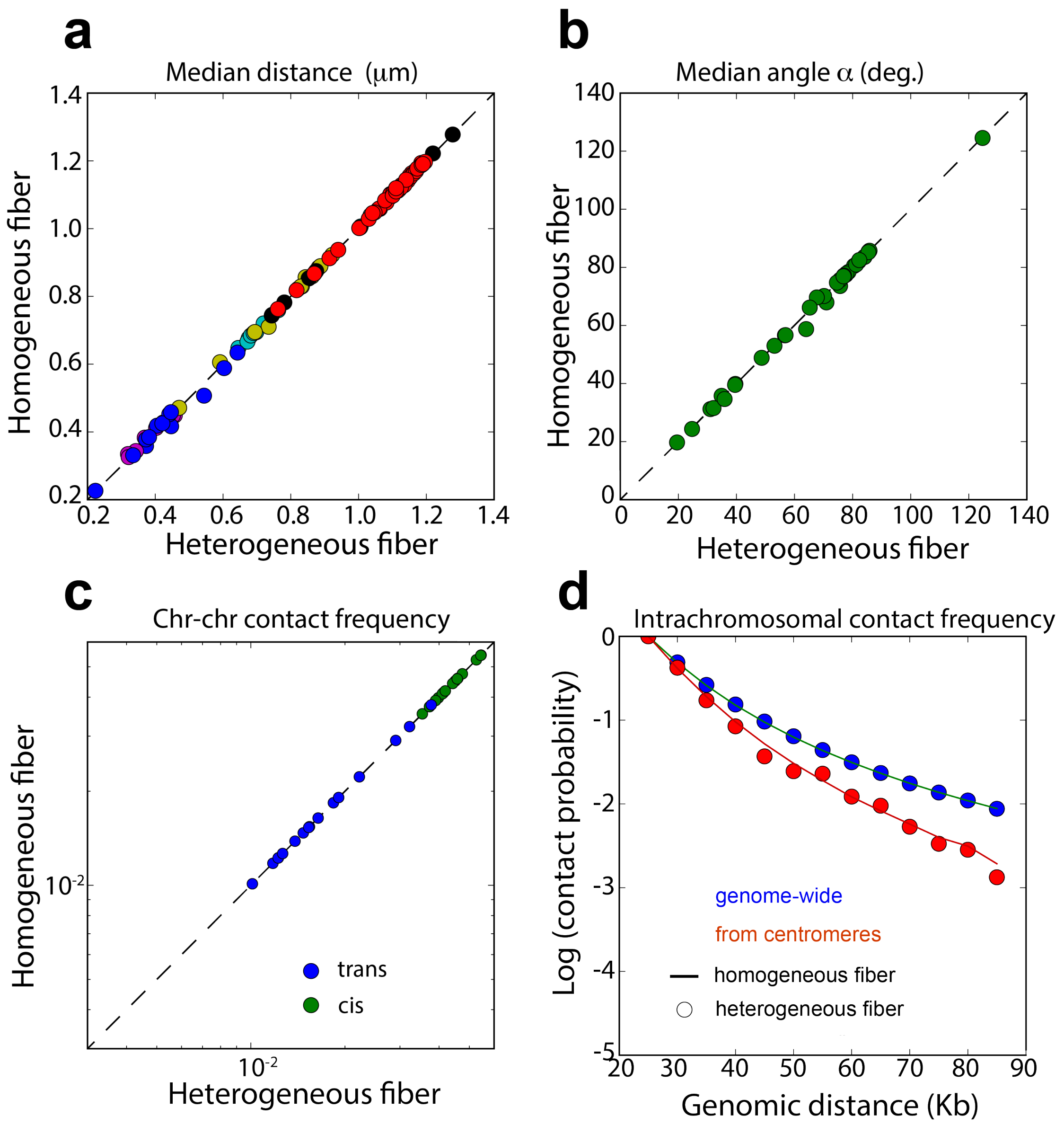

Figure S14: Predictions of heterogeneous vs homogeneous fiber models

These plots show observables predicted by a heterogeneous chromatin fiber model vs. a homogeneous chromatin fiber model. The models were defined as described for **Fig. S13**. **a**) median distances between pairs of loci (as in **Fig. 4a**). **b**) median angles between chromatin loci and the line joining the nuclear and nucleolar centers (as in **Fig. 4c**). **c**) Contact frequencies between pairs of chromosomes normalized by the product of their genomic lengths (as in **Fig. S10**). **d**) Intrachromosomal contact frequencies as function of the genomic distance averaged over the entire genome (blue) or centered on centromeres (red). Solid curves are from the homogeneous model, circles from the heterogeneous model.

Supplementary Methods

A. Langevin dynamics simulations

A.1 Chromosomal polymer chains

All simulations were run using the molecular dynamics software package LAMMPS[8] (http://lammps.sandia.gov). Each of the 16 yeast chromosomes was represented as a chain of spherical monomers (beads) connected by a harmonic potential (see below). The number of beads of a chromosome of genomic length L (in bp) is the closest integer to $L/(CW)$, where C is the compaction (in bp/nm) and W is the bead diameter, i.e. the width of the fiber. Depending on these parameters, the total number of beads in the simulation ranged from 1,966 to 16,202. The same bead size W was used for all chromosomes, except for the rDNA locus on chromosome 12, which contains 100-200 repeats of the rDNA gene, and was modeled using 150 beads of larger diameter, $W_{\text{rDNA}} > W$. In all core simulations (**Fig. 1a**, **Table 1, Additional File 2**), we set W_{rDNA}= 194 nm, such that the net volume occupied by the rDNA beads approximates V = 14% of the nuclear volume. Because of the space between distinct rDNA beads, the total volume of the nucleolar compartment formed by the rDNA amounts to about $2V$ ~ 30% of the nucleus. We used a different nucleolar volume $V \neq 14\%$ only when generating test data to assess inference robustness to parameter mismatch (**Fig. S4**).

A.2 Bonds between monomers, persistence length

Consecutive monomers of a chromosome chain $(i, i+1)$ were linked by a finite extensible non-linear elastic (FENE) bond defined by the energy potential:

$$E_{\text{FENE}}(i, i+1) = -\frac{1}{2} K R_0^2 \ln\left[1 - \left(\frac{r_{i,i+1}}{R_0}\right)^2\right] + 4\varepsilon\left[\left(\frac{\sigma}{r_{i,i+1}}\right)^{12} - \left(\frac{\sigma}{r_{i,i+1}}\right)^6\right] + \varepsilon$$

where $r_{i,i+1} = \|\mathbf{r}_{i+1} - \mathbf{r}_i\|$ is the distance between the centers of the two beads of position vectors $\mathbf{r}_i$ and $\mathbf{r}_{i+1}$(**Fig. S15a**) We set the parameters as follows: $K = 30/w^2$, $R_0 = w$, $\varepsilon = 1, \sigma = w$, where w was defined as:

- $w = W$ for pairs of monomers outside of the rDNA region

- $w = W_{\mathrm{rDNA}}$ for pairs of monomers within the rDNA region
- $w = (W + W_{\mathrm{rDNA}})/2$ for pairs of monomers spanning the boundaries of the rDNA region (i.e. monomer i is inside the rDNA but $i+1$ is outside, or vice-versa).

(In practice, we set *W* to unity, but scaled all other length scales accordingly.)

In order to adjust the rigidity of the chromosome fiber, we added a harmonic angle potential between all triplets of consecutive monomers $(i-1, i, i+1)$ - (except for beads in the rDNA locus):

$$E_{\mathrm{bending}}(i) = K_0(\theta_i)^2$$

where θ_i is the angle between $\mathbf{r}_{i+1} - \mathbf{r}_i$ and $\mathbf{r}_i - \mathbf{r}_{i-1}$ and the constant K_0 controls the rigidity (**Fig. S15b**). In order to relate this constant to the chromatin persistence length *P*, we simulated a single long chromosome with 612 beads in absence of confinement and computed *P* as: $P = -\frac{\langle r_{i,i+1} \rangle}{\ln\langle \cos\theta_i \rangle} = -\frac{0.94\,W}{\ln\langle \cos\theta_i \rangle}$, where the brackets $\langle . \rangle$ denote statistical average and the second identity results from the FENE potential above. We considered four values of K_0: [0, 0.5, 1, 2], for which the resulting persistence lengths *P* were 0.9*W*, 1.5*W*, 2.3*W* and 4.2*W*, respectively.

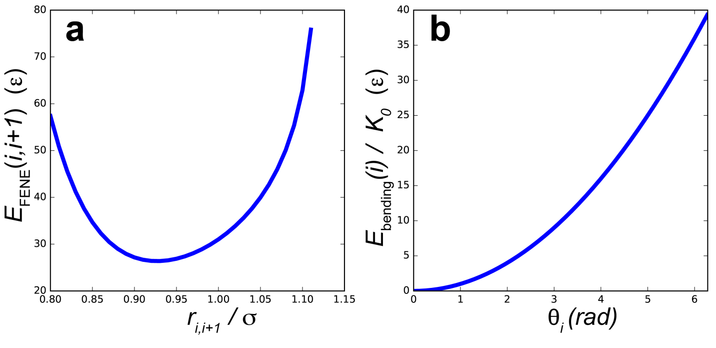

Figure S15: FENE and bending potentials

a) FENE potential as function of the normalized distance $r_{i,i+1}/\sigma = \|\mathbf{r}_{i+1} - \mathbf{r}_i\|/\sigma$ between consecutive monomers. **b**): Bending potential as function of the angle θ_i between consecutive bond vectors $\mathbf{r}_{i+1} - \mathbf{r}_i$ and $\mathbf{r}_i - \mathbf{r}_{i-1}$.

A.3 Repulsive interaction between arbitrary monomers

The interaction between non-consecutive monomers (i,j), $j \notin \{i-1, i, i+1\}$ is modeled with a truncated Lennard-Jones potential:

$$E_{\mathrm{LJ}}(i,j) = \begin{cases} 4\varepsilon\left[\left(\frac{\sigma}{r_{i,j}}\right)^{12} - \left(\frac{\sigma}{r_{i,j}}\right)^{6}\right] - 4\varepsilon\left[\left(\frac{\sigma}{r_0}\right)^{12} - \left(\frac{\sigma}{r_0}\right)^{6}\right] & if\ r_{i,j} \leq r_0 \\ 0 & if\ r_{i,j} > r_0 \end{cases}$$

where $r_{i,j} = \|\mathbf{r}_i - \mathbf{r}_j\|$ is the distance between the centers of the two monomers, $\varepsilon = 1$, r_0 is the cut-off distance, which was set to $r_0 = 1.15\sigma$ with $\sigma = (W_i + W_j)/2$, where W_i and W_j are the diameters of the beads i and j, respectively (**Fig. S16a**). The choice of $r_0 = 1.15\sigma$ creates a very small attractive potential for $r_{i,j}$ between 1.12σ and 1.15σ. However we verified that very similar results are obtained for $r_0 = 1.12\sigma$, as shown in **Fig. S4a,b** (red bars).

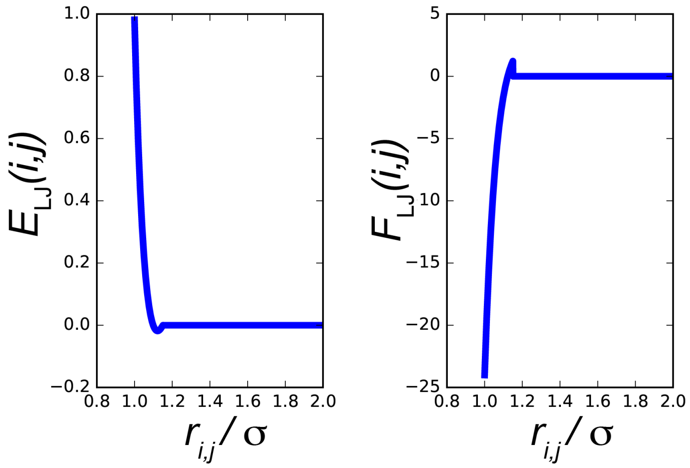

Figure S16: Repulsion between monomers.

Truncated and shifted Lennard-Jones potential (left) and the corresponding force (right) as function of the normalized distance $r_{i,j}/\sigma$ between the centers of monomer *i* and *j*. The truncation point is visible at around $r_{i,j}/\sigma = 1.12$

A.4 Nuclear confinement and tethering at telomeres

Confinement of the chromosomes by the spherical nuclear envelope was modeled using another truncated Lennard-Jones potential:

$$E_N(i) = \begin{cases} 4\varepsilon\left[\left(\frac{\sigma}{R_N - r_i}\right)^{12} - \left(\frac{\sigma}{R_N - r_i}\right)^{6}\right] - 4\varepsilon\left[\left(\frac{\sigma}{R_N - r_1}\right)^{12} - \left(\frac{\sigma}{R_N - r_1}\right)^{6}\right] & if\ r_i \geq R_N - r_1 \\ 0 & if\ r_{i,j} < R_N - r_1 \end{cases}$$

where $R_N - r_i$ is the distance between the center of bead i and the sphere of radius $R_N = 1000\ \mathrm{nm}$ representing the nuclear envelope; $\varepsilon = 1$; $\sigma = W_i$ is the bead diameter

($W_i = W$ for all beads except those in the rDNA locus, where $W_i = W_{\mathrm{rDNA}}$), and the cutoff distance is $r_1 = 1.12\, W_i$ (**Fig. S17a**). This potential was applied to all monomers of the simulation.

In order to account for the tethering of telomeres at the nuclear envelope[9], we added an attractive potential between each of the 32 beads at the extremities of the polymer chains, and the sphere of radius R_N:

$$E_{\mathrm{Tel}}(i) = \begin{cases} 4\varepsilon\left[\left(\frac{\sigma}{R_N - r_i}\right)^{12} - \left(\frac{\sigma}{R_N - r_i}\right)^{6}\right] - 4\varepsilon\left[\left(\frac{\sigma}{R_N - r_2}\right)^{12} - \left(\frac{\sigma}{R_N - r_2}\right)^{6}\right] & if\ r_i \geq R_N - r_2 \\ 0 & if\ r_{i,j} < R_N - r_2 \end{cases}$$

with $\varepsilon = 4$, $\sigma = W$ and $r_2 = 2\,W$ (**Fig. S17b**).

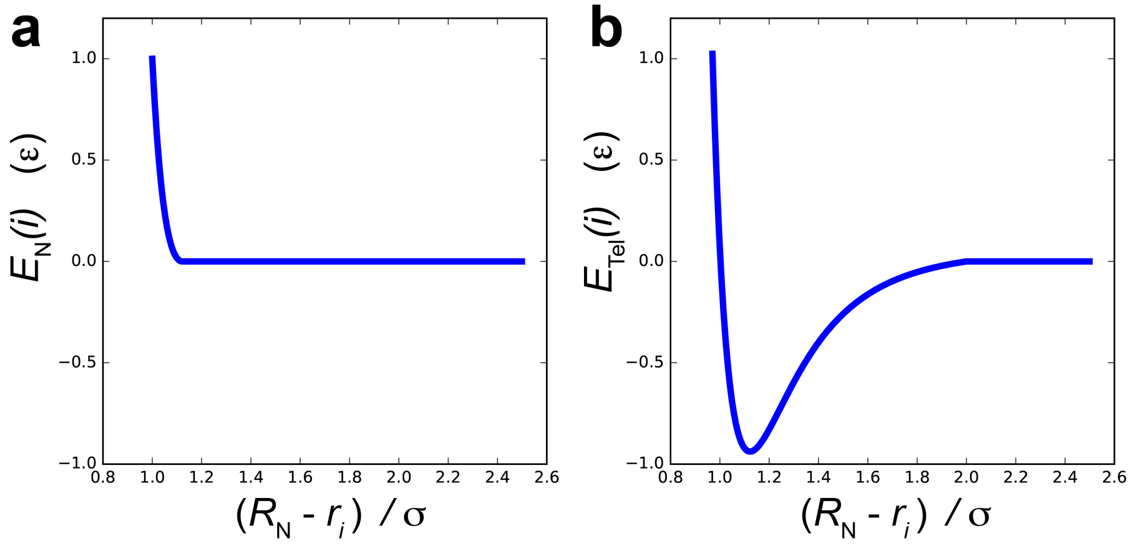

Figure S17: Potentials for nuclear confinement and telomeric tethering.

a) nuclear confinement potential $E_N(i)$. **b**) telomeric tethering potential $E_{\mathrm{Tel}}(i)$. In both cases, the potential is plotted as function of the normalized distance $\frac{R_N - r_i}{\sigma}$ of the monomer center to the nuclear envelope.

A.5 Centromeric tethering

To model the tethering of centromeres to the spindle pole body (SPB) via microtubules, we introduced a harmonic potential:

$$E_{\mathrm{cen}}(i) = K_{\mathrm{cen}}\left(r_{i,\mathrm{SPB}} - L\right)^2$$

where $r_{i,\mathrm{SPB}} = \|\mathbf{r}_i - R_N \mathbf{1}_x\|$ is the distance between the center of a centromeric bead and the SPB, and the spring constant was set to $K_{\mathrm{cen}} = 10$ (**Fig. S18**). The parameter L represents the length of the centromere-SPB link by microtubules and was varied from 200 to 400 nm (**Table 1, Additional File 2**). The value of K_{cen} was determined empirically to ensure that the average distance between centromere and SPB (which is also affected by other factors) remains close to L.

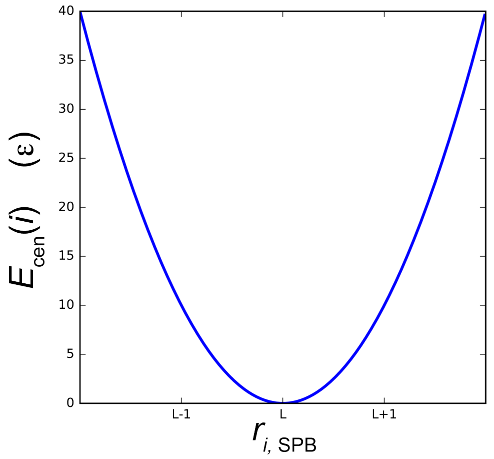

Figure S18: Centromeric tethering potential

The harmonic potential that tethers centromeres to the vicinity of the SPB, as function of the distance between the center of the centromeric bead and the SPB.

A.6 Langevin dynamics

The motion of a monomer is governed by the Langevin equation:

$$M_i \frac{d^2\mathbf{r}_i}{dt^2} = \mathbf{F}_i(t) - \zeta_i \frac{d\mathbf{r}_i}{dt} + \mathbf{R}_i(t)$$

where M_i is the monomer mass, $\mathbf{r}_i$ the monomer center position vector, $\mathbf{F}_i(t)$ is the deterministic force exerted on the monomer, ζ_i is the viscous damping coefficient, and $\mathbf{R}_i(t)$ is a random force of average zero $\langle \mathbf{R}_i(t) \rangle = 0$ and variance $\langle \mathbf{R}_i(t)^2 \rangle = 6k_B T \zeta_i$, where k_B is the Boltzmann constant and T is the temperature in Kelvins. In practice, the three components of $\mathbf{R}(t)$ are drawn from a Gaussian distribution with mean zero and variance $2k_B T \zeta_i$. We used reduced units, whereby the mass of each monomer was unity ($M_i = M_0 = 1$), except for monomers in the rDNA locus, where we used $M_i = M_{\mathrm{rDNA}} = 34$ (see below). The force $\mathbf{F}_i(t)$ was derived from the sum of the applicable potentials

described above:

$$\mathbf{F}_i(t) = -\nabla E(i)$$

For a generic monomer i (i.e. not a centromere, nor a telomere), we have:

$$E(i) = E_{\mathrm{FENE}}(i-1,i) + E_{\mathrm{FENE}}(i,i+1) + E_{\mathrm{bending}}(i) + \sum_{j \neq i} E_{\mathrm{LJ}}(i,j) + E_{\mathrm{N}}(i)$$

For a monomer corresponding to a centromere or telomere, the relevant potential $E_{\mathrm{cen}}(i)$ or $E_{\mathrm{Tel}}(i)$ was added to the right hand side of this equation.

The damping coefficient of non-rDNA monomers was set to: $\zeta_i = \zeta_0 = 1/2$ (LJ unit). For the larger rDNA monomers, we scaled the damping coefficient with the monomer radius, i.e. we set $\zeta_i = \zeta_0 (M_{\mathrm{rDNA}}/M_0)(W/W_{\mathrm{rDNA}})$. The mass of the rDNA beads, M_{rDNA}, was chosen based on the smallest ratio of bead diameters between rDNA and non-rDNA (W_{rDNA}/W) and found to be consistent with the observed differences in chromatin dynamics between rDNA loci and non-other loci as reported in ref. [10].

LAMMPS provides a numerical implementation of the Langevin equation, with discrete time intervals, which we set to 0.005.

A.7 Initialization and equilibration

The initial configuration of chromosomes was generated as a random walk, as shown in **Fig. S1a,b**. Then a confining sphere of radius $R_0 > R_N$ was introduced that encloses all chromosomes. During the first phase of the simulation, we progressively decreased this radius until it reached R_N. The force that tethers centromeres to the SPB ($-\nabla E_{\mathrm{cen}}$) was turned on only after this point.

Before using the model to predict various observables, we checked if the simulations are equilibrated. To assess equilibration, we run at least two, and up to six independent simulations with the same parameter values ('replicate simulations', **Additional File 2**), but with different (random) initializations (**Fig. S1a,b**). We used more replicates for the models with larger numbers of monomers. In cases with more than two replicates, we randomly assign them to two groups *A* and *B*. We then compare the mean predicted observables from these two groups. If the simulation is equilibrated, the two independent predictions should be similar. The specific observables we considered are the 62 average telomere-telomere distances[1] d_i , $i = 1..62$ (**O1, Table 2, Additional File 3**).

We call d_i^A and d_i^B the average distance for telomere-telomere pair i predicted by simulations from groups *A* and *B*, respectively. These predicted distances are plotted against each other in **Fig. S2a** for all 144 models, indicating very good agreement between replicates. For the vast majority of models, the relative difference $q = 100\times\frac{1}{62}\sum_{i=1}^{62}\frac{|d_i^A-d_i^B|}{(d_i^A+d_i^B)/2}$ was below 5% and in no case exceeded 7% (**Fig. S2b**). Thus, the independent replicate simulations yielded similar results for all simulations, therefore to a good approximation the simulations appear to be equilibrated.

The number of time steps in each simulation ranged from $8\text{x}10^8$ to $8\text{x}10^9$, depending on the number of monomers, as systems with more monomers need more time to equilibrate.

B. Predicting observables from simulations

B.1 Sampling the simulation

In order to predict observables listed in **Table 2** and **Additional File 3**, we considered the simulation trajectories after 10^8 time steps - i.e. at least 3 times the decorrelation time- and sampled the chromosome configurations every 10,000 time steps (see **Fig. S1c,d**). We then pooled these samples with those from the replicate simulation(s). Temporal statistics over simulation time were used to predict cell population statistics at a single time. For example, the predicted distances between two loci A and B averaged over samples from a single simulation run were compared to the distances between fluorescently tagged loci in a single microscopy snapshot, averaged over all cells in the image[1].

B.2 Predicting spatial distances

The prediction of 3D distance statistics from the simulation samples (as required for comparison with observables O1, O3, O5-O7 in **Table 2** and **Additional File 3**) is straightforward. To compute projected 2D distances (as required for observable O4) from pairs of 3D coordinates, we randomly dropped one of the three coordinates from each sample. To predict distances between telomeres and the SPB (7 data points, observable O7), we assumed that the SPB is located at the position $(x_{\text{SPB}}, y_{\text{SPB}}, z_{\text{SPB}}) = (-R_N - 70\text{ nm}, 0, 0) = (-1070\text{ nm}, 0, 0)$. The 70 nm shift was meant to account for the fact that the protein Spc42p -which was used to tag the SPB in the experimental

measurements[11]- is located roughly 70 nm from the nucleoplasmic face of the SPB, where microtubules attach [12], [13]. Since Spc42 is actually at ~20 nm outside the nuclear envelope, this choice could lead to an overestimate of predicted distances between Spc42 and chromatin loci located in close to the SPB. However, in practice, the distances between SPc42 and chromatin loci (all telomeres) in the experimental data used here all exceeded ~0.9 µm (**Table 2** and **Additional File 3**). Therefore, the 50 nm additional shift entails at most a ~5% error in predicted distances and does not significantly impact our results. We indeed verified that using $x_{\mathrm{SPB}} = -R_N - 20\ \mathrm{nm}$ has little consequence on the 7 predicted distances.

B.3 Predicting contact frequencies

Predicting contact frequencies from the simulation requires a definition of contact events. We scored a contact between two monomers i and j if the distance $r_{i,j}$ between their centers fell below a 'capture radius' r_c. The choice of r_c affects the predicted contact probabilities : on average, larger r_c increased interchromosomal contact probabilities more than intrachromosomal contact probabilities when these were normalized to one (see **Fig. S19a**). This introduces an additional unknown parameter r_c in the comparison between model predictions and experimental measurements. However the capture radius is known to affect predominantly contacts between genomically proximal loci[14]. Therefore, we ignored all intrachromosomal contacts between loci separated by less than 20 Kb. This restriction effectively removed the dependency of chromosomal contact probabilities on r_c (see **Fig. S19b**). In all our predictions, we used r_c=1.15 *W*.

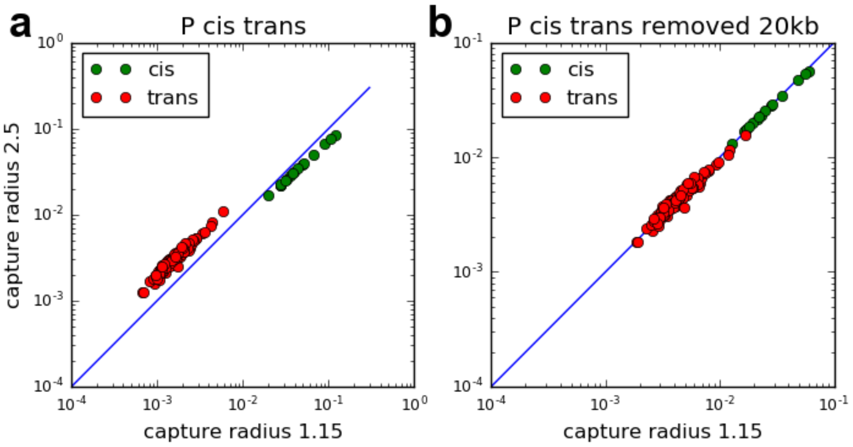

Figure S 19: Effect of capture radius on predicted contact probabilities

Both panels compare the contact probabilities between chromosomes predicted for two different

capture radii ($r_c = 2.5\,W$ vs $r_c = 1.15\,W$). Each of the 120 red dots corresponds to contacts between a distinct pair of chromosomes (*trans* contacts), each of the 16 green dots corresponds to contacts within a single chromosome (*cis* contacts). In **a**), all contacts are considered, in **b**) *cis* contacts between loci are ignored if their genomic separation is 20 Kb or less. Note that in all cases, the contact frequencies were normalized to probabilities, such that the sum of all 120 interchromosomal and 16 intrachromosomal probabilities equals one. Simulation parameters were *P* = 54 nm, *C* = 83 bp/nm, *W* = 60 nm, *L* = 400 nm (model #44 in **Additional File 2**).

Using the above capture radius, each time point of the simulation was turned into a binary contact matrix of size *n x n*, where *n* is the number of monomers in the model. Summing all binary matrices from the sampled simulation run yielded a predicted contact frequency matrix (**Fig. 4d**) that can be compared to Hi-C data (see **Fig. 4e,f**). For our parameter inference method, rather than using the full resolution contact frequency matrix as input, we chose to use the following two sets of summary statistics:

- Contacts frequencies between chromosomes in *cis* and *trans*: if N_{tot} is the total number of scored contacts across the genome (counting contacts between monomers (k,l) and (l,k) only once), N_i the total number of contacts involving chromosome i ($i = 1..16$), L_i the genomic length of chromosome i, $N_{cis,i}$ the number of contacts within chromosome i, and $N_{trans,i} = N_i - N_{cis,i}$ the number of contacts of chromosome i with other chromosomes, then we defined: $p_{cis,i} = N_{cis,i}/\left(L_i^2 N_{tot}\right)$ and $p_{trans,i} = N_{trans,i}/\left(2L_i \sum_{j\neq i} L_j\, N_{\mathrm{tot}}\right)$ as the *cis*-and *trans*-chromosomal contact probabilities, normalized by the genomic lengths of the chromosomes. The 32 numbers $p_{cis,i}$ and $p_{trans,i}$ ($i = 1..16$) provide the first set of statistics comprising our contact frequency observables in O8 and O9 (**Table 2**).
- Intrachromosomal contact frequencies: As a second set of statistics, we considered average intrachromosomal contact frequencies as function of genomic distance. Specifically, we discretized genomic distances in 5 Kb intervals from 25 to 85 Kb, thereby defining 12 genomic bins $[s_k, s_{k+1}]$, $k = 0..11$ ($s_0 = 25\,Kb$; $s_{k+1} = s_k + 5\,Kb$). We defined $N_{\mathrm{all},k}$ as the total number of contacts between any of the 16 centromeres and monomers separated by a genomic distance $s \in [s_k, s_{k+1}[$. We also define $N_{\mathrm{cen},k}$ as the number of contacts between pairs of monomers separated by a genomic distance $s \in [s_k, s_{k+1}[$. The corresponding probabilities were then obtained after

normalizing to the value at 25 Kb, i.e. we computed $p_{\text{all},k} = N_{\text{all},k}/N_{\text{all},0}$ and $p_{cen,k} = N_{\text{cen},k}/N_{\text{cen},0}$ $k = 0..11$. Finally, because these probabilities fall off rapidly with genomic distance[2], [7], we replaced them by their logarithm, $l_{\text{all},k} = \ln p_{\text{all},k} = \ln(N_{\text{all},k}/N_{\text{all},0})$ and $l_{\text{cen},k} = \ln p_{\text{cen},k} = \ln(N_{\text{cen},k}/N_{\text{cen},0})$. These probabilities provided 24 additional data points from contact frequencies for observables O8 and O9 (**Table 2**).

Together, $p_{cis,i}$, $p_{trans,i}$, $l_{\text{all},k}$ and $l_{\text{cen},k}$ thus provide 56 predicted contact probabilities (or their logarithm) that can be compared to the 56 experimental measurements.

B.4 Interpolating predictions

Sections B.2, B.3, and B.4 above described how various observables are predicted from our simulations for a given value of the parameters Π_i, where $i \in [1,144]$ (**Additional File 2**). We denote $M_i = M(\Pi_i)$ the model corresponding to parameter value Π_i and $Y_k^{M_i}$ the prediction by this model of observable k, where $k \in [1,266]$ (see **Table 2**). For each observable k, our inference method requires computing its predicted values $Y_k^{M(\Pi)}$ for arbitrary, continuous values of the parameters Π (within the range spanned by the extreme values, see **Table 1**). Therefore an interpolation scheme is needed to compute $Y_k^{M(\Pi)}$ from the discrete set of values $Y_k^{M_i}$, $i = 1..144$. We used radial basis function interpolation as implemented in the Python scipy library, with a linear kernel and the smoothing factor set to 50 for observables O1, 10 for O2-O7, and 0 for O8 and O9.

C. Bayesian inference and priors

C.1 Bayes rule and likelihood

As described in the main text, our inference method computes the posterior probability density of the parameters Π from the experimental data D using the Bayes rule:

$$p(\Pi|D) \propto p(D|\Pi)p(\Pi)$$

where $p(D|\Pi)$ is the likelihood and $p(\Pi)$ is the prior on the parameters.

The data D consist in one or more of the data sets O1-O9 described above. Each data set, for example O1, consists of multiple data points, $D = (Y_1^E, \dots, Y_N^E)$. We note the corresponding model predictions as $(Y_1^M, \dots, Y_N^M)$. The likelihood expresses the probabilistic relationship between measurements Y_i^E and model predictions Y_i^M. For

simplicity, we assume that each predicted value is related to its corresponding measurements via a Gaussian probability density, whose mean and variance σ_i^2 depend on the data set as described below. Specifically, we assume that:

$$p(D|\Pi) = p(Y_1^E|\Pi)\times \dots .\times p(Y_N^E|\Pi)$$

$$= \frac{1}{\sqrt{2\pi}\sigma_1}\exp\left(-\frac{(Y_1^E - [Y_1^M + B_1])^2}{2\sigma_1^2}\right)\dots .\frac{1}{\sqrt{2\pi}\sigma_N}\times\exp\left(-\frac{(Y_N^E - [Y_N^M + B_N])^2}{2\sigma_N^2}\right)$$

where B_i accounts for the possible presence of a bias (see below). In general, the variance σ_i^2 depends on the nature and value of the variable Y_i^E (e.g. the distance between loci or contact frequency between chromosomes, etc.) and on additional unknown parameters, called nuisance parameters, generically noted Ξ. The bias B_i depends on the prediction Y_i^M as well as the nuisance parameter Ξ. The specific forms of these relationships will be detailed for each type of observable below. We treat the nuisance parameter Ξ as additional parameters to be inferred along with the structural parameters Π, thus reformulating the Bayes rule as:

$$p(\Pi, \Xi|D) \propto p(D|\Pi, \Xi)p(\Pi)p(\Xi)$$

Therefore, three quantities must be computed to evaluate the posterior probability $p(\Pi, \Xi|D)$ (up to a normalization factor): (i) the likelihood $p(D|\Pi, \Xi)$, (ii) the prior on the structural parameters, $p(\Pi)$ and (iii) the prior on the nuisance parameters $p(\Xi)$.

- The likelihood is evaluated as:

$$p(D|\Pi, \Xi) = p(Y_1^E|\Pi, \Xi)\times \dots .\times p(Y_N^E|\Pi, \Xi)$$

$$= \frac{1}{\sqrt{2\pi}\sigma_1(\Xi, Y_1^E)}\times\exp\left(-\frac{(Y_1^E - [Y_1^M + B_1(\Xi, Y_1^M)])^2}{2\sigma_1^2(\Xi, Y_1^E)}\right)\dots .\frac{1}{\sqrt{2\pi}\sigma_N(\Xi, Y_1^E)}$$

$$\times\exp\left(-\frac{(Y_N^E - [Y_N^M + B_N(\Xi, Y_1^M)])^2}{2\sigma_N^2(\Xi, Y_1^E)}\right)$$

where we have emphasized the dependency of σ_i and B_i on the measurements Y_i^E , the predictions Y_i^M and the nuisance parameter(s) Ξ. The specific forms of the likelihood for each type of data will be detailed below.

- The prior on the structural parameters is assumed to be flat, i.e.: $p(\Pi) = p(P, C, W, L) = p(P)p(C)p(W)p(L) = \text{constant}$ (over the range of parameter values indicated in **Table 1**).

- The prior on the nuisance parameter(s), $p(\Xi)$, depends on the type of

observable, and will be described below.

C.2 Likelihood and nuisance prior for distances between loci

For observables O1, O3-O7, each data point Y_i^E is the mean, median or mode of distances between two loci. We assume that Y_i^E is related to its predicted counterpart Y_i^M by a Gaussian probability density:

$$p\left(Y_i^E \middle| \Pi, n\right) = \frac{1}{\sqrt{2\pi}\sigma(n)} \exp\left(-\frac{\left(Y_i^E - Y_i^M\right)^2}{2\sigma^2(n)}\right)$$

where the variance is given by $\sigma \quad (n) = Y_i^E/\sqrt{n}$, and n is a normally distributed random variable with mean and standard deviation 1,000 (the order of magnitude of the number of cells analyzed in some of the distance data sets, e.g. [1]).

Combining all data points within a given set of data $D = (Y_1^E, \ldots, Y_N^E)$ (e.g. all data points in O1), we obtain the following likelihood:

$$p_{\text{distances}}(D|\Pi, n) = \left(\frac{n}{2\pi}\right)^{N/2} \frac{1}{Y_1^E \times \cdots \times Y_N^E} \exp\left(-\frac{(Y_1^E - Y_1^M)^2}{2(Y_1^E)^2} n\right) \times \cdots \times \exp\left(-\frac{(Y_N^E - Y_N^M)^2}{2(Y_N^E)^2} n\right)$$

and the prior density on the nuisance parameter n is:

$$p_{\text{distances}}(n) = \frac{1}{\sqrt{2\pi \times 1000^2}} \exp\left(-\frac{(n-1000)^2}{2 \times 1000^2}\right)$$

C.3 Likelihood and nuisance prior for angles of nuclear locus territories

Observable O2 contains median angles α between loci, nuclear and nucleolar centers[1], [15]. Random errors in the experimental measurements of these three points lead to a substantial systematic overestimation of these angles. To account for this bias, we assumed the following relation between measured and predicted median angles Y_i^E and Y_i^M:

$$p_{\text{angles}}\left(Y_i^E \middle| \Pi, \sigma_\alpha, \sigma_{\text{Nuc}}\right) = \frac{1}{\sqrt{2\pi}\sigma_\alpha} \exp\left(-\frac{\left(Y_i^E - \left[Y_i^M + B\left(Y_i^M, \sigma_{\text{Nuc}}\right)\right]\right)^2}{2\sigma_\alpha^2}\right)$$

Here, σ_{Nuc} represents the standard deviation of localization errors of the nucleolar center, and $B\left(Y_i^M, \sigma_{\text{Nuc}}\right)$ the bias in measured angles due to these errors. In practice, we compute this bias numerically as $B\left(Y_i^M, \sigma_{\text{Nuc}}\right) = Y_i^{M\prime}(\sigma_{\text{Nuc}}) - Y_i^M$, where $Y_i^{M\prime}(\sigma_{\text{Nuc}})$ are

median angles obtained from the simulation after addition of random localization errors of standard deviation σ_{Nuc} to the nucleolar center. Based on a previous estimate of these errors[15], we assume the following prior for the nuisance parameter σ_{Nuc}:

$$p_{\text{angles}}(\sigma_{\text{Nuc}}) = \frac{1}{\sqrt{2\pi}\times(10\text{ nm})}\exp\left(-\frac{(\sigma_{\text{Nuc}}-30\text{ nm})^2}{2\times(10\text{ nm})^2}\right)$$

and for the nuisance parameter σ_α, we further assume:

$$p_{\text{angles}}(\sigma_\alpha) = \frac{1}{\sqrt{2\pi}\times(5\text{ deg.})}\exp\left(-\frac{(\sigma_{\text{Nuc}}-10\text{ deg.})^2}{2\times(5\text{ deg.})^2}\right)$$

Taking into account all angle measurements $D = (Y_1^E, \ldots, Y_N^E)$ in O2, the likelihood is then expressed as:

$$p_{\text{angles}}(D|\Pi,\sigma_\alpha,\sigma_{\text{Nuc}}) = \left(\frac{1}{\sqrt{2\pi}\sigma_\alpha}\right)^N \exp\left(-\frac{\left(Y_1^E-[Y_1^M+B(Y_1^M,\sigma_{\text{Nuc}})]\right)^2}{2\sigma_\alpha^2}\right)\times\cdots\times$$
$$\exp\left(-\frac{\left(Y_N^E-[Y_N^M+B(Y_N^M,\sigma_{\text{Nuc}})]\right)^2}{2\sigma_\alpha^2}\right)$$

C.4 Likelihood and noise prior for contact frequencies

As discussed above (section B.3), we summarize the contact frequency data into two sets of probabilities comprising: (i) 2x16=32 probabilities of contacts within and between chromosomes ($p_{cis,i}$ and $p_{trans,i}$, collectively denoted below as $p_i, i = 1..32$), (ii) 2x12=24 log-probabilities of intrachromosomal contacts for 12 genomic bins, either averaged over the genome ($l_{\text{all},k}$) or restricted to the centromeres ($l_{\text{cen},k}$), here collectively noted $l_i, i = 1..24$. We separately discuss the p_i and l_i below:

The probabilities p_i are obtained as ratios of the number of contacts between two chromosomes N_i and the total number of contacts N_{tot}, i.e. $p_i = N_i/N_{\text{tot}}$. The number of contacts N_i obeys a multinomial distribution, which for large N_{tot} can be approximated by a Gaussian distribution of mean $p_i N_{\text{tot}}$ and variance $p_i(1-p_i)N_{\text{tot}}$. The probability p_i then follows a Gaussian distribution of mean p_i and variance $p_i(1-p_i)/N_{\text{tot}}$.

In theory, N_{tot} is directly provided by the experimental data and is thus not a random number. However, in practice, several additional factors besides counting noise can affect the measured contact frequencies[16]–[18]. This is evidenced by a comparison of both Hi-C data sets [3], [4], for which the measured probabilities differ by up to 10%

despite a large total number of contacts N_{tot}. In order to accommodate additional sources of noise in a simple manner, we therefore instead assume that N_{tot} follows a Gaussian distribution of mean 100 and standard deviation 100. Under these assumptions the likelihood for all 32 contact probabilities $D = (Y_1^E, \dots, Y_{32}^E)$ taken together becomes:

$$p_{\text{chr-chr contacts}}(D|\Pi, n) = \left(\sqrt{\frac{n}{2\pi}}\right)^{32} \frac{1}{\sqrt{Y_1^E(1-Y_1^E)}\times\cdots\sqrt{Y_{32}^E(1-Y_{32}^E)}} \exp\left(-\frac{(Y_1^E-Y_1^M)^2}{2Y_1^E(1-Y_1^E)}n\right)\times$$
$$\cdots \exp\left(-\frac{(Y_{32}^E-Y_{32}^M)^2}{2Y_{32}^E(1-Y_{32}^E)}n\right)$$

with the prior on the nuisance parameter n given by:

$$p_{\text{chr-chr contacts}}(n) = \frac{1}{\sqrt{2\pi\times100^2}} \exp\left(-\frac{(n-100)^2}{2\times100^2}\right)$$

Concerning the 24 log-probabilities l_i of intrachromosomal contacts as function of genomic distance, we assume that they obey the following Gaussian probability distribution:

$$p_{\text{intrachr contacts}}(D|\Pi, \sigma) = \left(\frac{1}{\sqrt{2\pi\sigma}}\right)^{32} \exp\left(-\frac{(Y_1^E - Y_1^M)^2}{2\sigma^2}\right)\times\cdots\times\exp\left(-\frac{(Y_{24}^E - Y_{24}^M)^2}{2\sigma^2}\right)$$

with the following prior on the nuisance parameter σ:

$$p_{\text{intrachr contacts}}(\sigma) = \frac{1}{\sqrt{2\pi}0.3} \exp\left(-\frac{(\sigma-0.3)^2}{2\times(0.3)^2}\right)$$

D. Generation of synthetic validation data

The synthetic data used to validate the inference method were obtained from our simulations. We first used simulations among our ‘core simulations’, which were used in the inference method itself (**Fig. 2**). For the robustness analysis, we also used simulations with parameters outside the range used in the core simulations (**Fig. S4**). The simulation was sampled to predict observables as described above, then we added three levels of random noise ('low', 'medium' and 'high') as specified below for different observables:

- For the mean, median, or mode of distances between loci (**Table 2**, observables O1, O3-7), we added Gaussian noise of mean 0 and standard deviation σ^S =0.3 nm (low noise), 30 nm (medium noise) or 60 nm (high noise). The largest noise level was chosen based on previous estimates of random errors in locus positioning and distance measurements [1].
- For the median angles between loci, nuclear center and nucleolar centroid[1], [15] (**Table 2,** observable O2), we added Gaussian noise of mean 0 and standard deviation of 0 (low noise), 1 (medium noise), or 5 degrees (high noise).
- For the contact probabilities between chromosomes, p_i (see sections B.3 and C.4), we added Gaussian noise of mean 0 and standard deviation equal to the probability multiplied by 0.03% (low noise), 5% (medium noise) or 10% (high noise). This was done as an approximation of counting noise, which necessarily affects contact frequencies determined from a finite number of ligation events by Hi-C[18]. We discarded negative values and repeated the addition of noise from the predicted frequency until the result was positive. We then renormalized the noisy values to a sum of 1 to obtain probabilities. The highest noise level was chosen to be higher than the difference between two independent Hi-C data sets[3], [4], for which we found that chromosomal contact probabilities differed on average by 2 % and at maximum by 7 %.
- For the log-probabilities of intrachromosomal contact as a function of genomic distance, l_i (see sections B.3 and C.4), we added Gaussian noise of mean 0 and standard deviation equal to l_i multiplied by 0.03% (low noise), 5% (medium noise) or 10% (high noise).

E. Genome-wide contact frequency data

We used four different experimental data sets on genome-wide contact frequencies, obtained by four distinct groups: a Hi-C data set from Duan et al. [4], a Hi-C data set from Marie-Nelly et al. [3], a Hi-C data set from Belton et al.[5], and a Micro-C XL data set from Hsieh et al. [6].

- For the Hi-C data of Duan et al. [4] we used contact frequency data from http://noble.gs.washington.edu/proj/yeast-architecture/sup.html . Specifically, we combined contact frequency data from Hi-C experiments using HindIII and EcoRI, as

given by the following four files:

- "interactions_HindIII_MseI_beforeFDR_inter.txt"
- "interactions_HindIII_MseI_beforeFDR_intra_all.txt"
- "interactions_EcoRI_MspI_beforeFDR_inter.txt"
- "interactions_EcoRI_MspI_beforeFDR_intra_all.txt"

- For the Hi-C data of Marie-Nelly et al. [3], we used contact frequency data from file 'Supplementary-S_cerevisiae_raw_matrix.dat' available at http://bioinformatics.oxfordjournals.org/content/30/15/2105/suppl/DC1
- For the Hi-C data of Belton et al.[5], we used contact frequency matrices from the file 'GSM1905065_yHiC-yJB1-R1_sk1-rmv_genome_H-10000-iced.matrix.gz-n2r.txt.gz' available at: https://www.ncbi.nlm.nih.gov/geo/query/acc.cgi?acc=GSM1905065
- For the Micro-C XL data of Hsieh et al. [6], we considered the experimental condition corresponding to a 3% formaldehyde concentration and a DSG crosslinker and generated the contact frequency matrix after processing the paired-end sequencing file (Run SRR4000664) available at:

  https://www.ncbi.nlm.nih.gov/sra?term=SRX2000979

F. Intrachromosomal distances on chromosome 4

Our analyzed data contain a subset of previously unpublished measurements of intrachromosomal distances. These data consist in 12 distributions of distances between pairs of loci on the right arm of chromosome 4. The 12 pairs of loci were chosen in distinct regions of the chromosome arm (within the pericentromeric region or in the internal region of the chromosome arm), and the distance between the loci varied between 40 and 231 Kb. For each of the 12 pairs, we constructed a yeast strain with one locus fluorescent labelled in green by insertion of 128 Lac operators and expression of LacI-eGFP, and the other labelled in red by insertion of 256 Tet operators and expression of TetR-mRFP. Cells were imaged live with a 60x oil immersion objective with a numerical aperture of 1.4 on a Nikon widefield microscope and using an Andor Neo sCMOS camera. We acquired 3D z-stacks of 35 frames with z-steps of 300 nm. Using a dual band filter set (eGFP, mRFP), we acquired two color channels

consecutively for each z position with an exposure time of 100 ms. 3D stacks were projected in 2D by maximum-intensity projection and distances between the red and green loci were measured automatically on manually selected cells using a custom-made Fiji plugin. Several hundreds of cells were analyzed for each of the 12 pairs of loci. Example distributions are shown in **Figure S8a-e**. More details on these data will be provided in an upcoming manuscript (Herbert et al., in revision).

Supplementary references:

[1] P. Thérizols, T. Duong, B. Dujon, C. Zimmer, and E. Fabre, “Chromosome arm length and nuclear constraints determine the dynamic relationship of yeast subtelomeres,” *Proc. Natl. Acad. Sci.*, vol. 107, no. 5, p. 2025, 2010.

[2] H. Wong, H. Marie-Nelly, S. Herbert, P. Carrivain, H. Blanc, R. Koszul, E. Fabre, and C. Zimmer, “A Predictive Computational Model of the Dynamic 3D Interphase Yeast Nucleus.,” *Curr. Biol.*, vol. 22, no. 20, pp. 1881–90, Oct. 2012.

[3] H. H. Marie-Nelly, M. Marbouty, A. Cournac, G. Liti, G. Fischer, C. Zimmer, and R. Koszul, “Filling annotation gaps in yeast genomes using genome-wide contact maps.,” *Bioinformatics*, vol. 30, no. 15, pp. 2105–2113, Apr. 2014.

[4] Z. Duan, M. Andronescu, K. Schutz, S. McIlwain, Y. J. Kim, C. Lee, J. Shendure, S. Fields, C. A. Blau, and W. S. Noble, “A three-dimensional model of the yeast genome,” *Nature*, vol. 465, no. 7296, pp. 363–367, May 2010.

[5] J. M. Belton, B. R. Lajoie, S. Audibert, S. Cantaloube, I. Lassadi, I. Goiffon, D. Baù, M. A. Marti-Renom, K. Bystricky, and J. Dekker, “The Conformation of Yeast Chromosome III Is Mating Type Dependent and Controlled by the Recombination Enhancer,” *Cell Rep.*, vol. 13, no. 9, pp. 1855–1867, Dec. 2015.

[6] T.-H. S. Hsieh, G. Fudenberg, A. Goloborodko, and O. J. Rando, “Micro-C XL: assaying chromosome conformation from the nucleosome to the entire genome,” *Nat. Methods*, Oct. 2016.

[7] E. Lieberman-Aiden, N. L. van Berkum, L. Williams, M. Imakaev, T. Ragoczy, A. Telling, I. Amit, B. R. Lajoie, P. J. Sabo, M. O. Dorschner, R. Sandstrom, B. Bernstein, M. A. Bender, M. Groudine, A. Gnirke, J. Stamatoyannopoulos, L. a Mirny, E. S. Lander, and J. Dekker, “Comprehensive mapping of long-range interactions reveals folding principles of the human genome.,” *Science*, vol. 326, no. 5950, pp. 289–93, Oct. 2009.

[8] S. Plimpton, “Fast Parallel Algorithms for Short-Range Molecular Dynamics,” *J. Comput. Phys.*, vol. 117, no. 1, pp. 1–19, 1995.

[9] C. Zimmer and E. Fabre, “Principles of chromosomal organization: lessons from yeast,” *J Cell Biol*, vol. 192, no. 5, pp. 723–733, 2011.

[10] B. Albert, J. Mathon, A. Shukla, H. Saad, C. Normand, I. Léger-Silvestre, D. Villa, A. Kamgoue, J. Mozziconacci, H. Wong, C. Zimmer, P. Bhargava, A. Bancaud,

and O. Gadal, “Systematic characterization of the conformation and dynamics of budding yeast chromosome XII.,” *J. Cell Biol.*, vol. 202, no. 2, pp. 201–10, Jul. 2013.

[11] K. Bystricky, T. Laroche, G. van Houwe, M. Blaszczyk, and S. M. Gasser, “Chromosome looping in yeast: telomere pairing and coordinated movement reflect anchoring efficiency and territorial organization,” *J Cell Biol*, vol. 168, no. 3, pp. 375–387, 2005.

[12] S. L. Jaspersen and M. Winey, “The budding yeast spindle pole body: structure, duplication, and function.,” *Annu. Rev. Cell Dev. Biol.*, vol. 20, pp. 1–28, Jan. 2004.

[13] E. T. O’Toole, M. Winey, and J. R. McIntosh, “High-voltage electron tomography of spindle pole bodies and early mitotic spindles in the yeast Saccharomyces cerevisiae,” *Mol Biol Cell*, vol. 10, no. 6, pp. 2017–2031, 1999.

[14] A. Rosa, N. B. Becker, and R. Everaers, “Looping probabilities in model interphase chromosomes,” *Biophys J*, vol. 98, no. 11, pp. 2410–2419, 2010.

[15] A. B. Berger, G. G. Cabal, E. Fabre, T. Duong, H. Buc, U. Nehrbass, J. C. Olivo-Marin, O. Gadal, and C. Zimmer, “High-resolution statistical mapping reveals gene territories in live yeast,” *Nat. Methods*, vol. 5, no. 12, pp. 1031–1037, 2008.

[16] A. Cournac, H. Marie-Nelly, M. Marbouty, R. Koszul, and J. Mozziconacci, “Normalization of a chromosomal contact map,” *BMC Genomics*, vol. 13, no. 1, p. 436, Aug. 2012.

[17] E. Yaffe and A. Tanay, “Probabilistic modeling of Hi-C contact maps eliminates systematic biases to characterize global chromosomal architecture,” *Nat Genet*, vol. 43, no. 11, pp. 1059–1065, 2011.

[18] M. Imakaev, G. Fudenberg, R. P. McCord, N. Naumova, A. Goloborodko, B. R. Lajoie, J. Dekker, and L. A. Mirny, “Iterative correction of Hi-C data reveals hallmarks of chromosome organization.,” *Nat. Methods*, vol. 9, no. 10, pp. 999–1003, Oct. 2012.
